# Characterisation of mineral deposition systems associated with rock art in the Kimberley region of northwest Australia

**DOI:** 10.1016/j.dib.2017.08.029

**Published:** 2017-09-07

**Authors:** Helen Green, Andrew Gleadow, Damien Finch

**Affiliations:** The School of Earth Sciences, The University of Melbourne, Australia

**Keywords:** Mineralogy, Accretions, X-ray diffraction, Laser ablation, Scanning electron microscopy, Rock art

## Abstract

This data article contains mineralogical and chemical data from mineral accretions sampled from rock art shelters in the Kimberley region of north west Australia. The accretions were collected both on and off pigment and engraved rock art of varying styles observed in the Kimberley with an aim of providing a thorough understanding of the formation and preservation of such materials in the context of dating [1]. This contribution includes processed powder X-ray Diffraction data, Scanning Electron Microscopy energy dispersive spectroscopy data, and Laser Ablation ICP-MS trace element mapping data.

**Specifications Table**

**Table:** 

Subject area	*Archaeological Science*
More specific subject area	*Geochemistry of rock art shelters*
Type of data	*Table and Figures*
How data was acquired	1. ***Powder X-ray diffraction (XRD) analysis** (Bruker D8 Advance x-ray powder diffractometer with Ni-filtered Cu kα radiation (1.54* *Å), Department of Chemical and Biomolecular Engineering, The University of Melbourne),* 2. ***Scanning Electron Microscopy- Energy Dispersive X-ray Spectroscopy analysis (SEM-EDS)** (Quanta FEG 200 ESEM and a Phillips FEI XL30 environmental scanning electron microscope (ESEM) equipped with an OXFORD INCA energy-dispersive x-ray spectrometer (EDS), The School of Earth Sciences, The University of Melbourne)* 3. ***Laser Ablation-Inductively Coupled Plasma-Mass Spectrometry (LA-ICP-MS)** (Agilent 7700x quadrupole mass spectrometer, coupled to a Lambda Physik Compex UV 193* *nm excimer laser system, The School of Earth Sciences, The University of Melbourne)*
Data format	*Raw and analysed*
Experimental factors	*Characterisation of mineralogy and chemistry of mineral accretions associated with Kimberley rock art*
Experimental features	*Analysis of minerals, their quantities and their chemical composition*
Data source location	*Inland and coastal sites of the Kimberley region of Western Australia. Specific locations of the rock art sites from which samples were collected are not disclosed in this study in order to protect sites from unauthorised visitation and to respect the wishes of our indigenous partners. However, site localities relating to samples are given a reference number that correlates to an access-controlled archaeological site catalogue held at The University of Western Australia's Centre for Rock Art Research + Management.*
Data accessibility	*The data are available within this article*

**Value of the data**

• Data presented here will be useful to other researchers as a benchmark for X-ray diffraction analysis of the range of mineral accretion systems present in Kimberley rock art shelters
• Laser-Ablation trace element maps coupled with site and sample photographs of four distinct mineral systems present in shelters in the Kimberley region of Western Australia may be used to aid sampling strategies associated with future rock art dating programs

## 1 Data

### 1.1 Data from laser-ablation trace element mapping

**Fig. 1:**
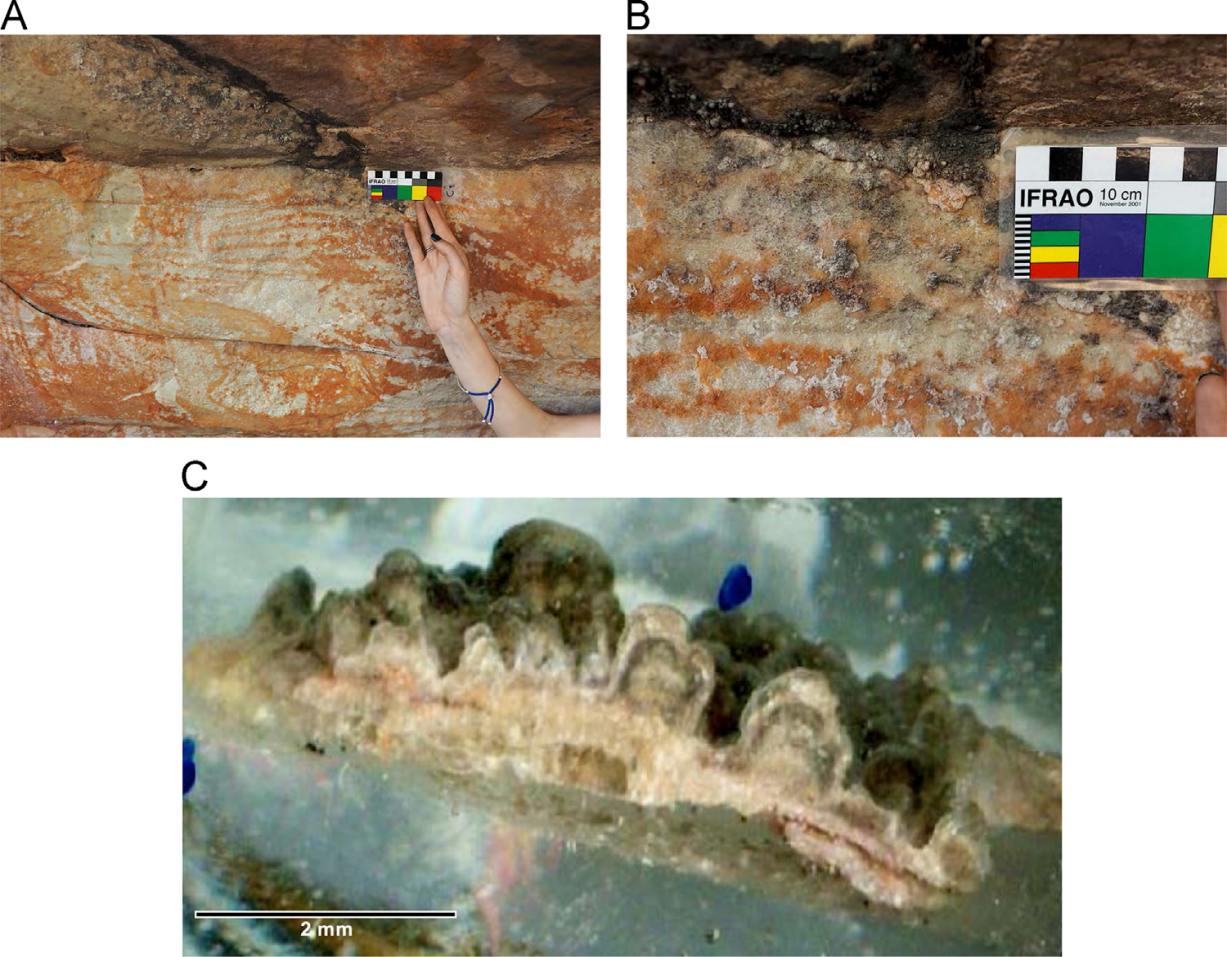
**(A)** Collection of sample H231 from a polychrome fringe mineral system on the wall of site D031 in the Drysdale River National Park region, **(B)** Polychrome fringe displaying distinctive banding and botryoidal, black surface of accretion sample H231 prior to removal, **(C)** Cross sectioned mount of polychrome accretion sample displaying a detailed internal stratigraphy.

**Fig. 2:**
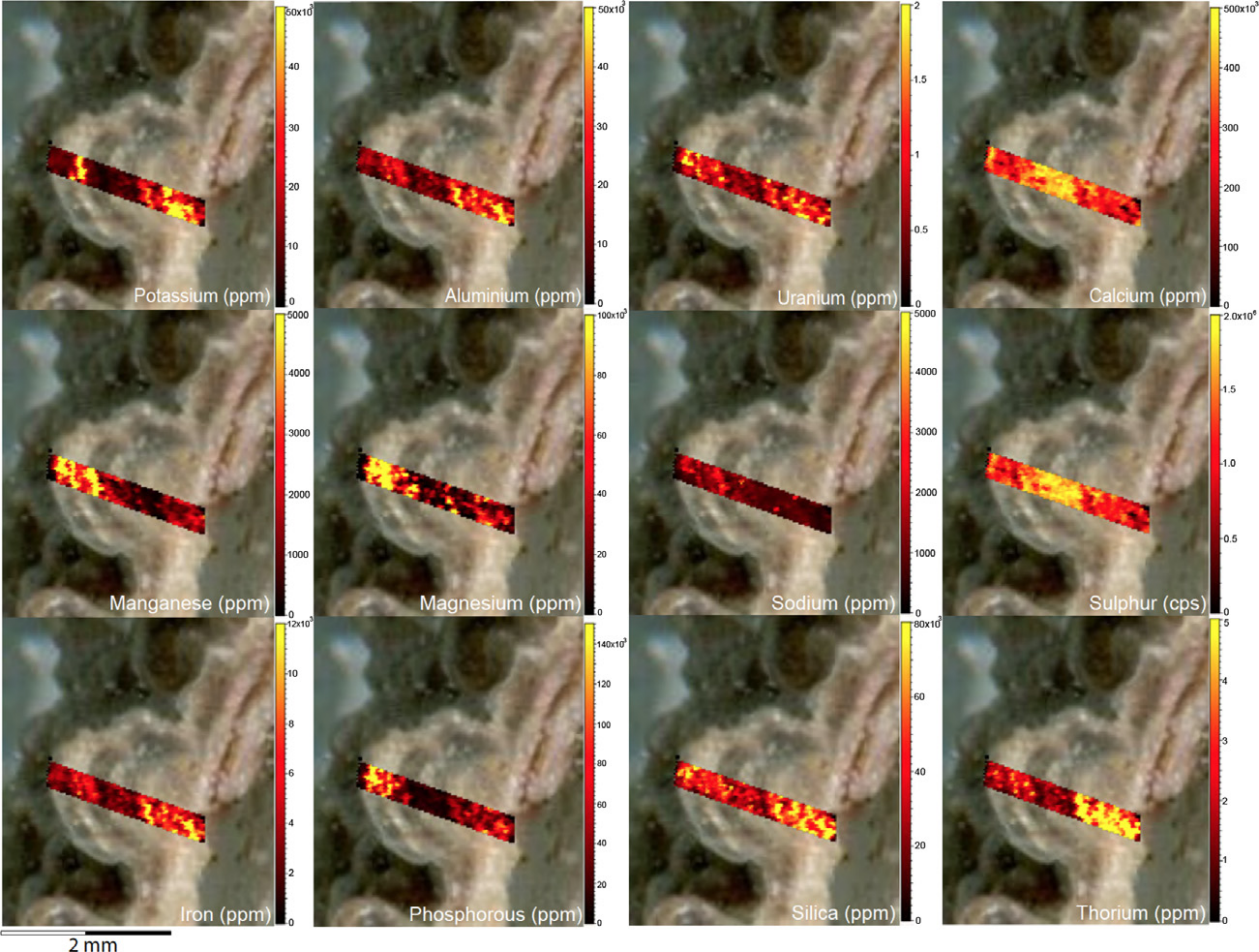
LA-ICP-MS trace element scans of polychrome fringe sample H231, sampled at site D031 in the Drysdale River National Park region. The scans display the distinct layering present in the botryoidal accretions characteristic of this mineral system.

**Fig. 3:**
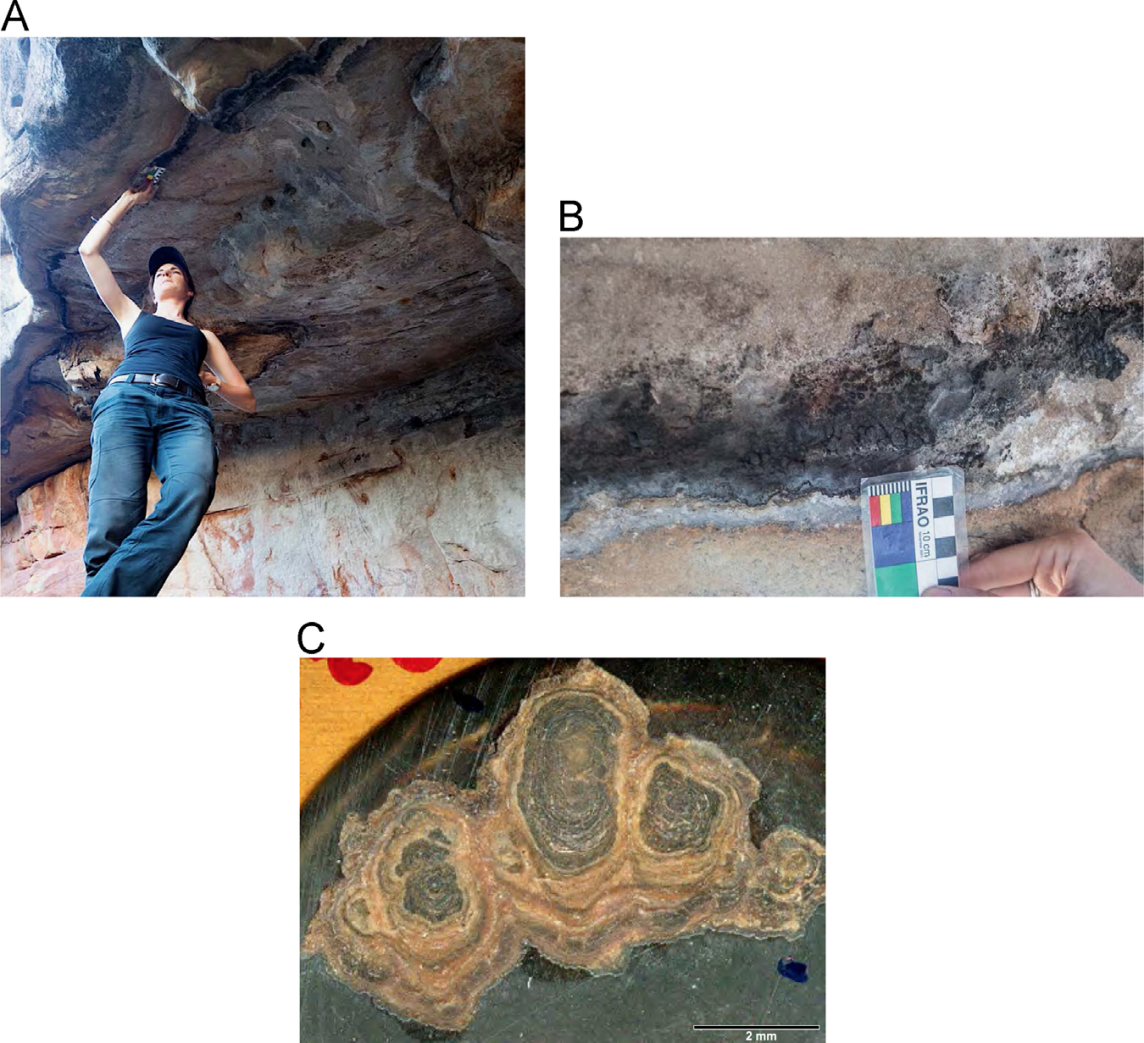
**(A)** Collection of sample H269 from a polychrome fringe mineral system on the ceiling of site D08 in the Drysdale River National Park region, **(B)** Polychrome fringe displaying distinctive banding and botryoidal, black surface of accretion sample H269 prior to removal, **(C)** Cross sectioned mount of polychrome accretion sample displaying a detailed internal stratigraphy.

**Fig. 4:**
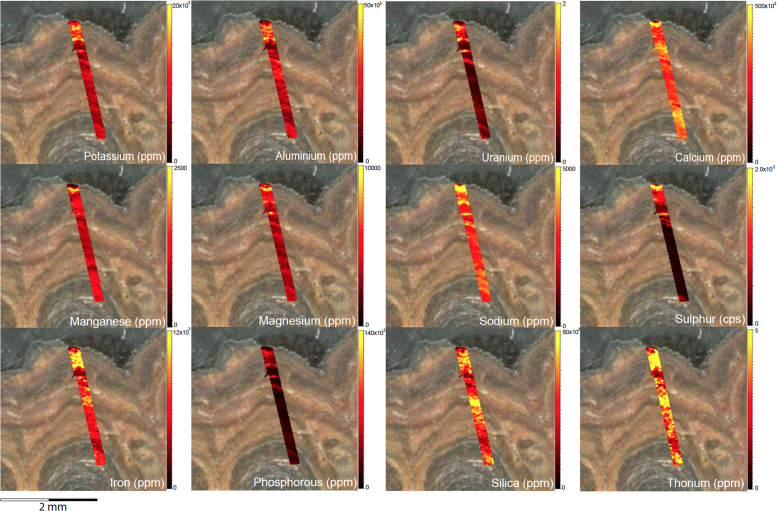
LA-ICP-MS trace element scans of polychrome fringe sample H269, sampled at site D08 in the Drysdale River National Park region. The scans display the distinct layering present in the botryoidal accretions characteristic of this mineral system.

**Fig. 5:**
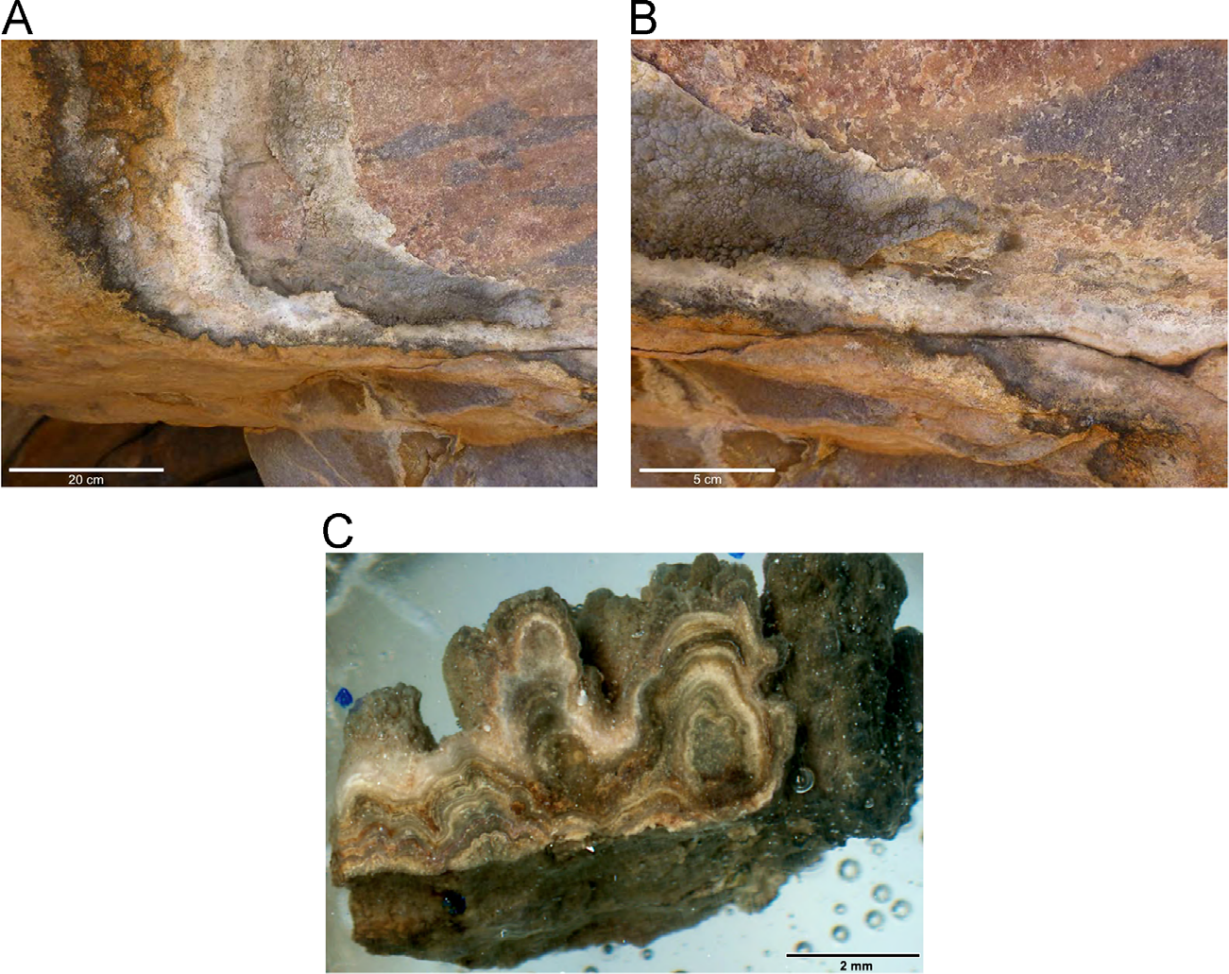
**(A)** Collection of sample OM14-15 from a polychrome fringe mineral system on the ceiling of site BL005 in the King George River region, **(B)** Polychrome fringe displaying distinctive banding and botryoidal, black surface of accretion sample OM14-15 prior to removal, **(C)** Cross sectioned mount of polychrome accretion sample OM14-15 displaying a detailed internal stratigraphy.

**Fig. 6:**
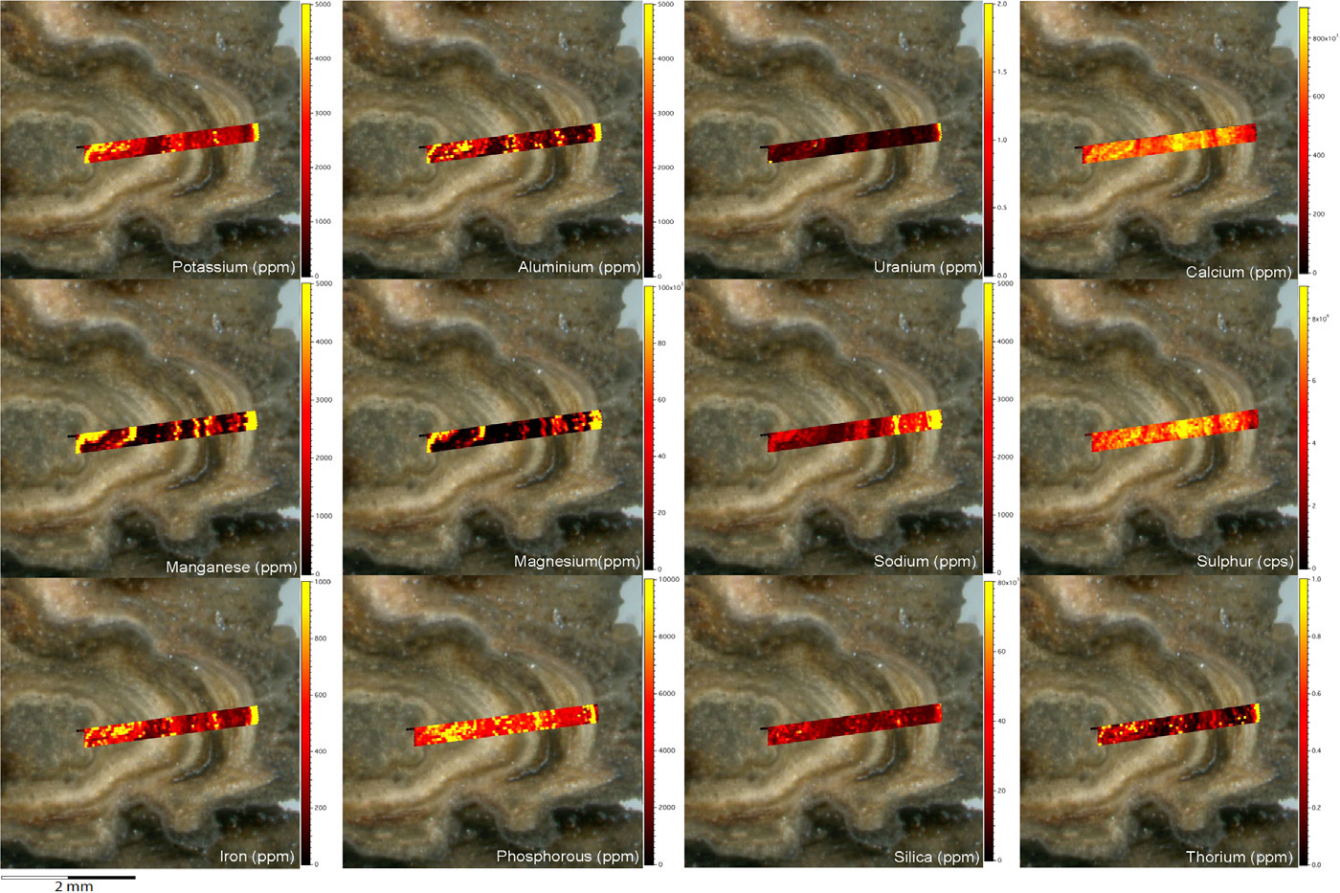
LA-ICP-MS trace element scans of polychrome fringe sample OM14-15, sampled at site BL005 in the King George River region. The scans display the distinct layering present in the botryoidal accretions characteristic of this mineral system.

**Fig. 7:**
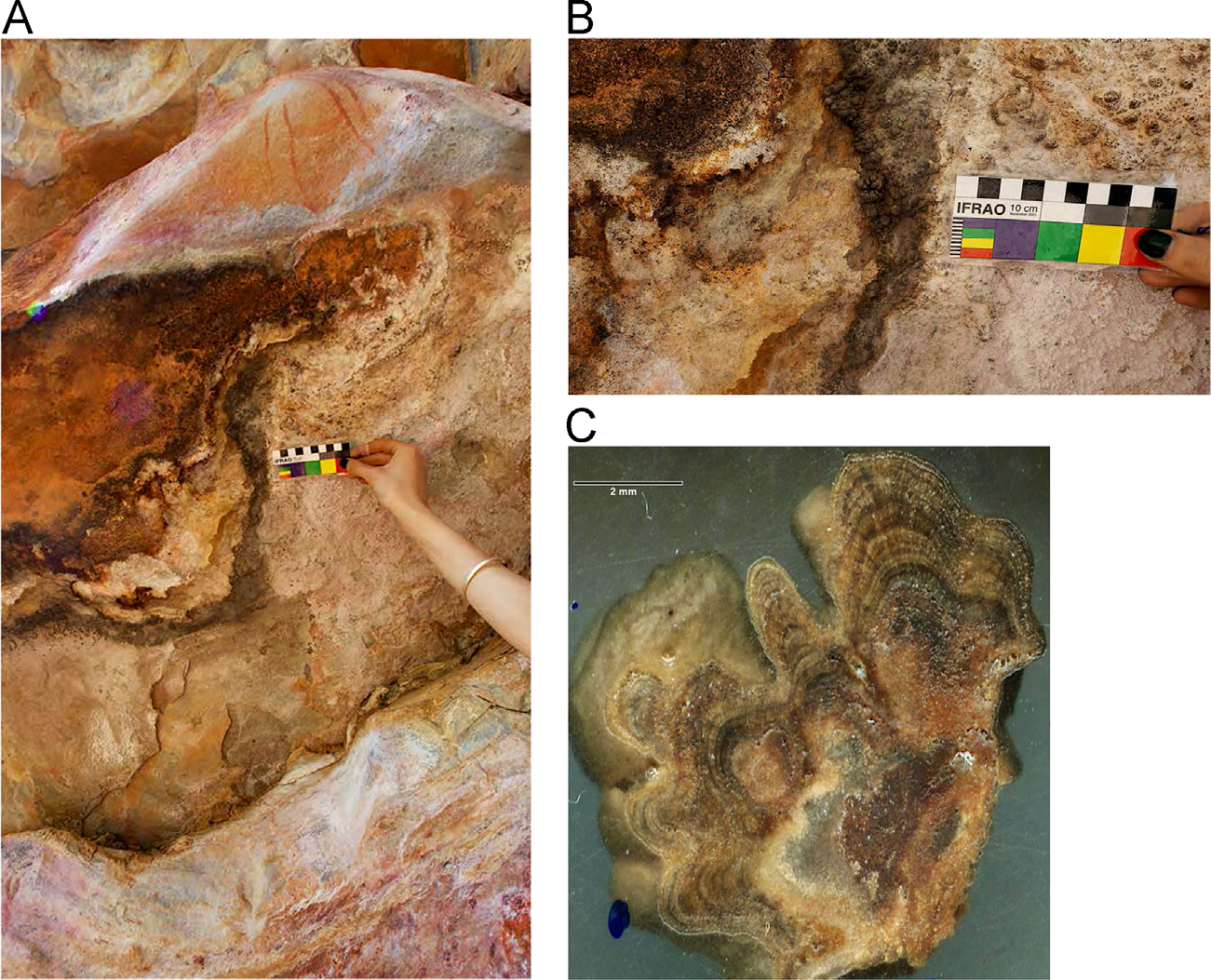
**(A)** Collection of sample H221 from a polychrome fringe mineral system on the ceiling of site DRY017 in the Drysdale River National Park region, **(B)** Polychrome fringe displaying distinctive banding and botryoidal, black surface of accretion sample H221 prior to removal, **(C)** Cross sectioned mount of polychrome fringe accretion sample H221 displaying a detailed internal stratigraphy.

**Fig. 8:**
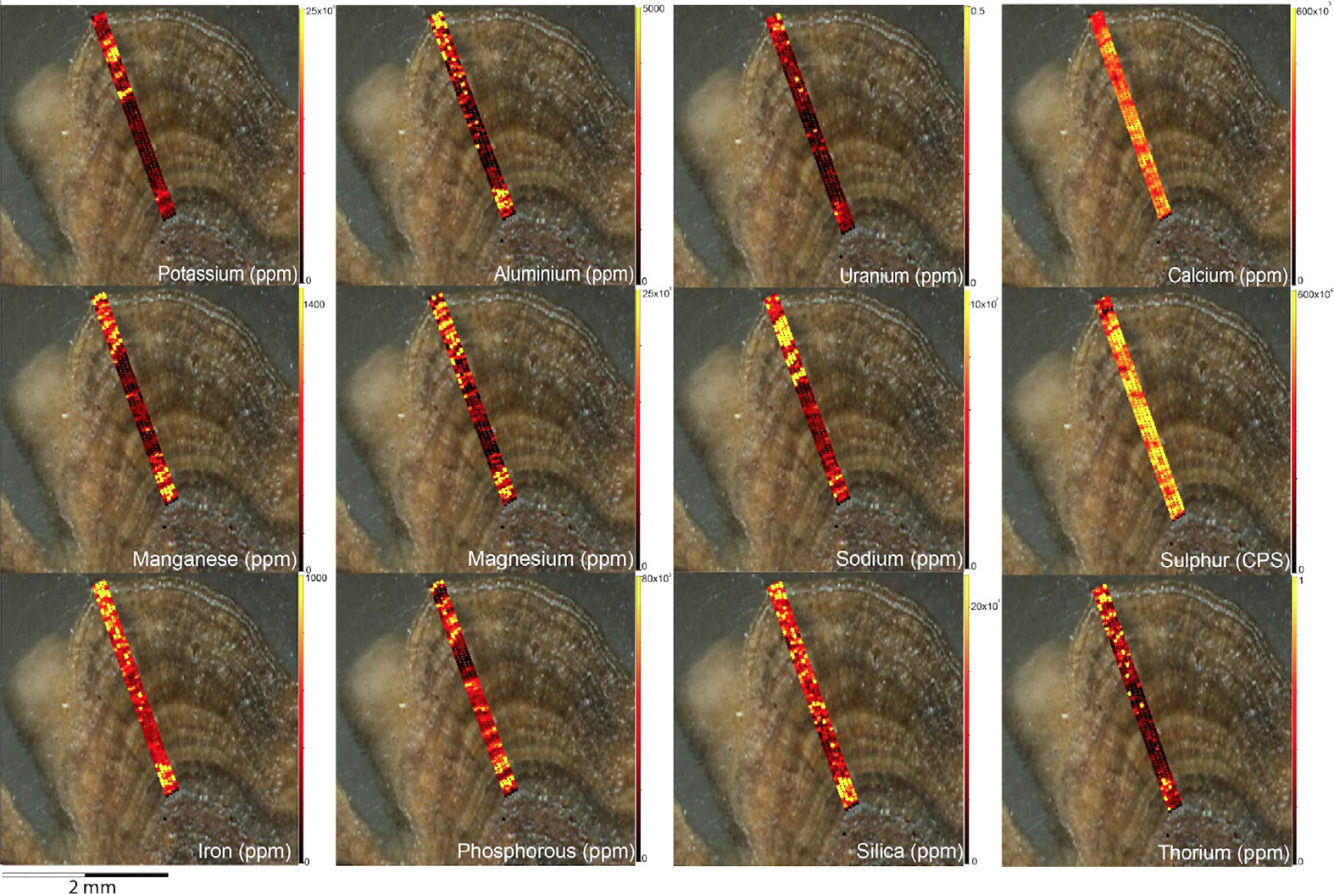
LA-ICP-MS trace element scans of polychrome fringe sample H221. The scans display the distinct layering present in the botryoidal accretions characteristic of this mineral system.

**Fig. 9:**
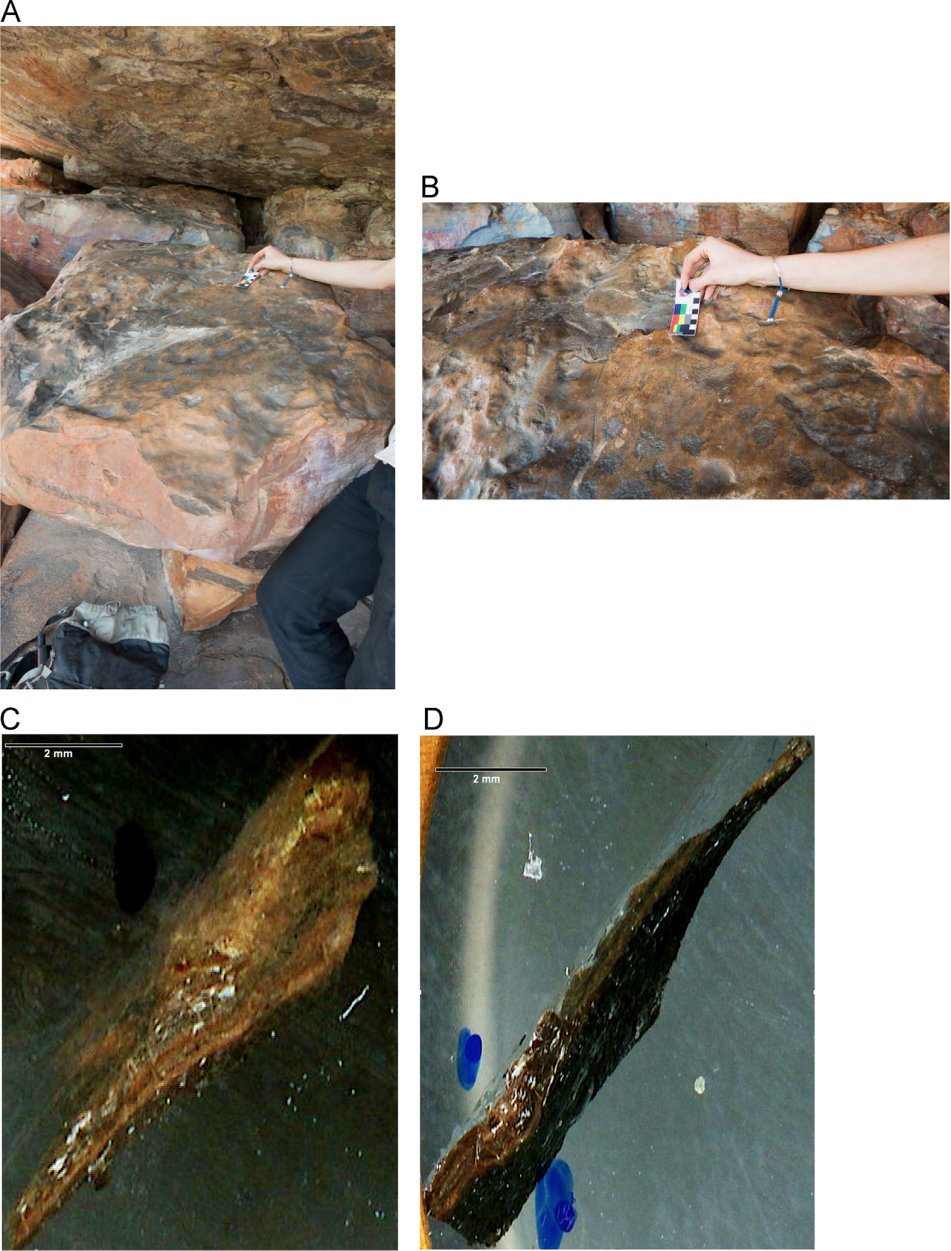
**(A)** Collection of sample H207 from a floor glaze mineral system covering a boulder in site D06 in the Drysdale River National Park region, **(B)** Floor glaze displaying distinctive smooth and dark coloured surface of accretion sample H207 with evidence of cupules ground into the surface, **(C and D)** Cross section mounts of floor glaze accretion sample H207 displaying an internal stratigraphy of thin, continuous layering.

**Fig. 10:**
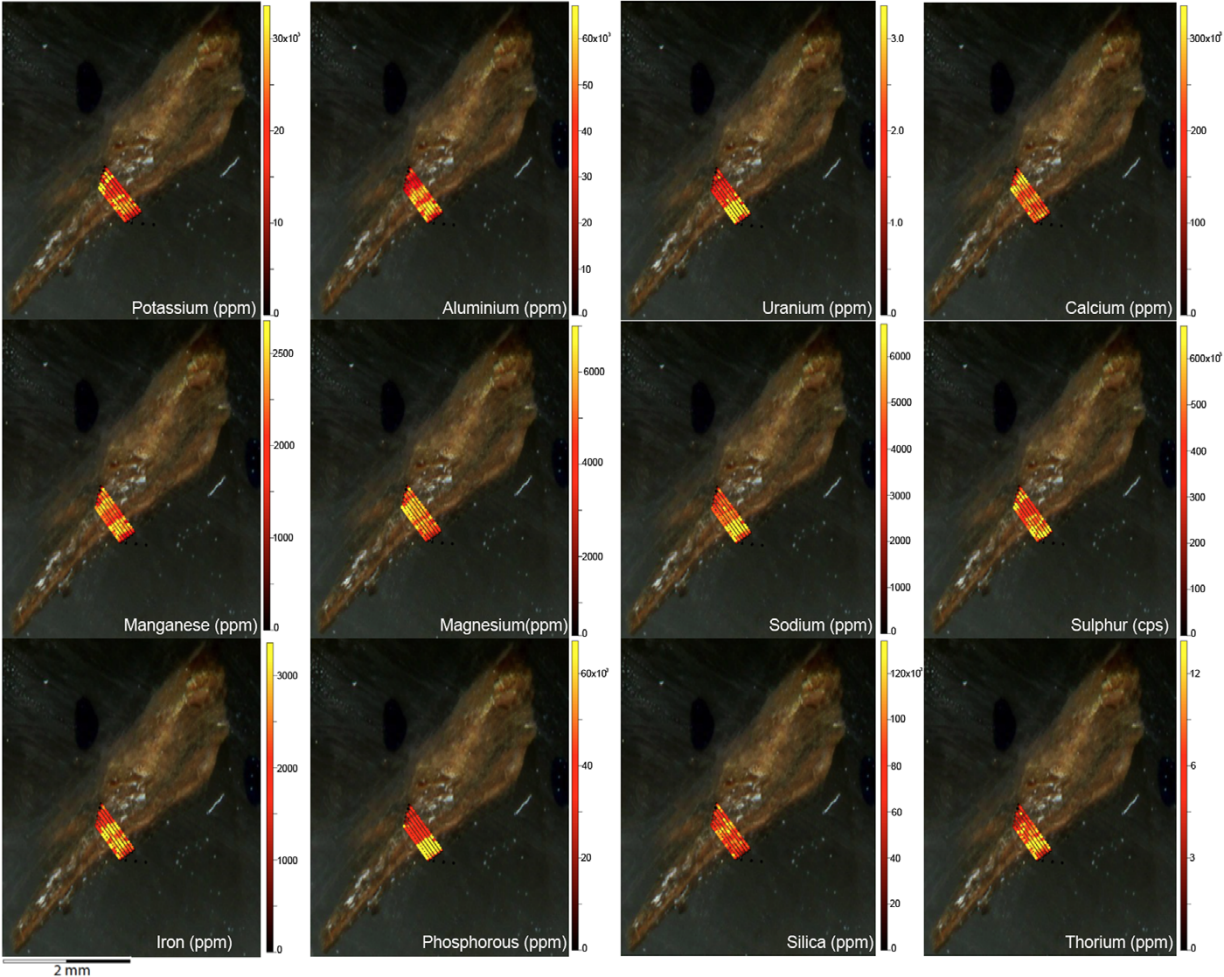
LA-ICP-MS trace element scans of floor glaze sample H207. The scans display the layering present in the floor glaze accretion, characteristic of this mineral system.

**Fig. 11:**
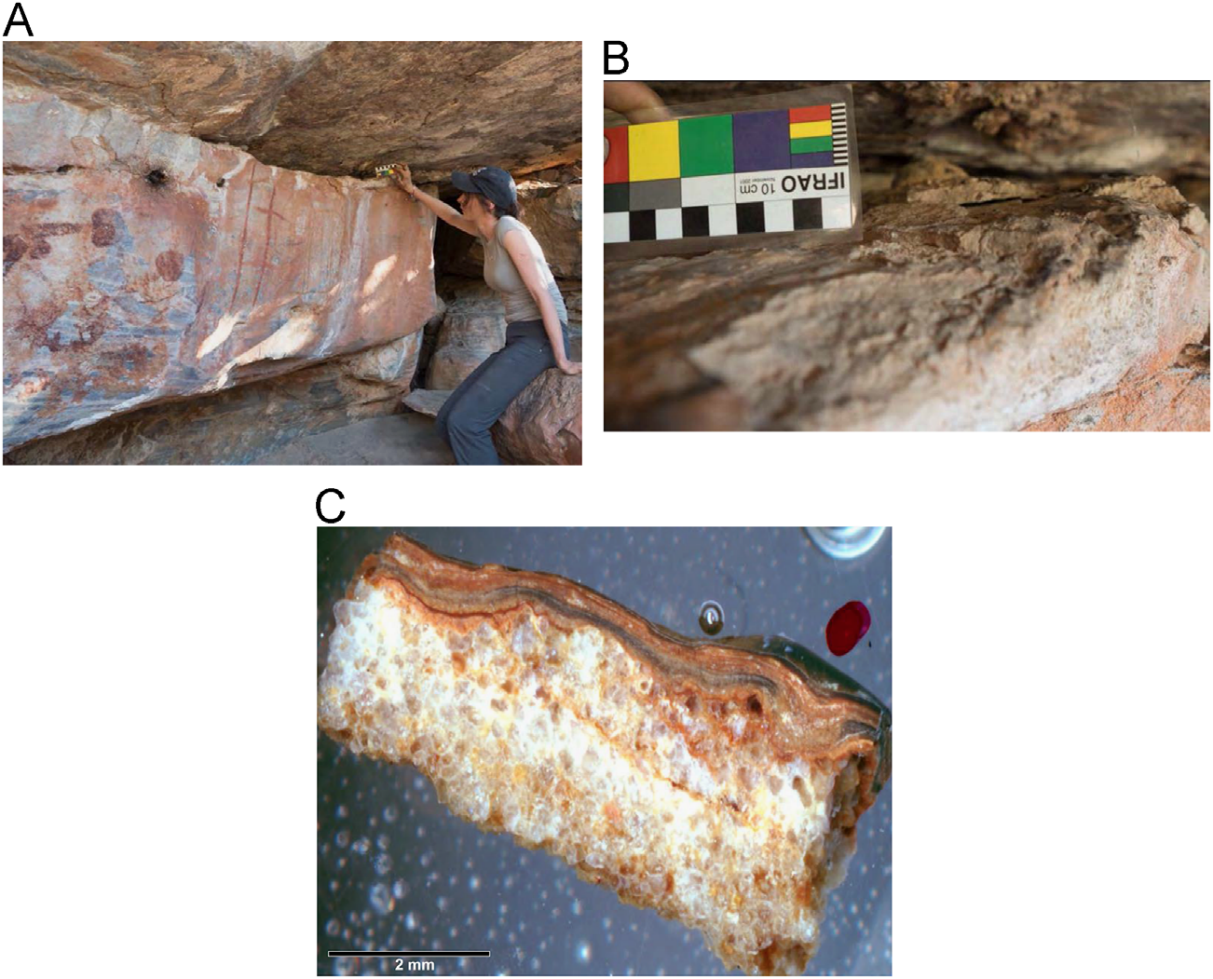
**(A)** Collection of sample H309 from a floor glaze mineral system in a deep ledge above a back wall in of site D050 in the Drysdale River National Park region, animals could have accessed this area **(B)** Floor glaze accretion sample H309 prior to removal, **(C)** Cross sectioned mount of floor glaze accretion sample H309 displaying a detailed internal stratigraphy.

**Fig. 12:**
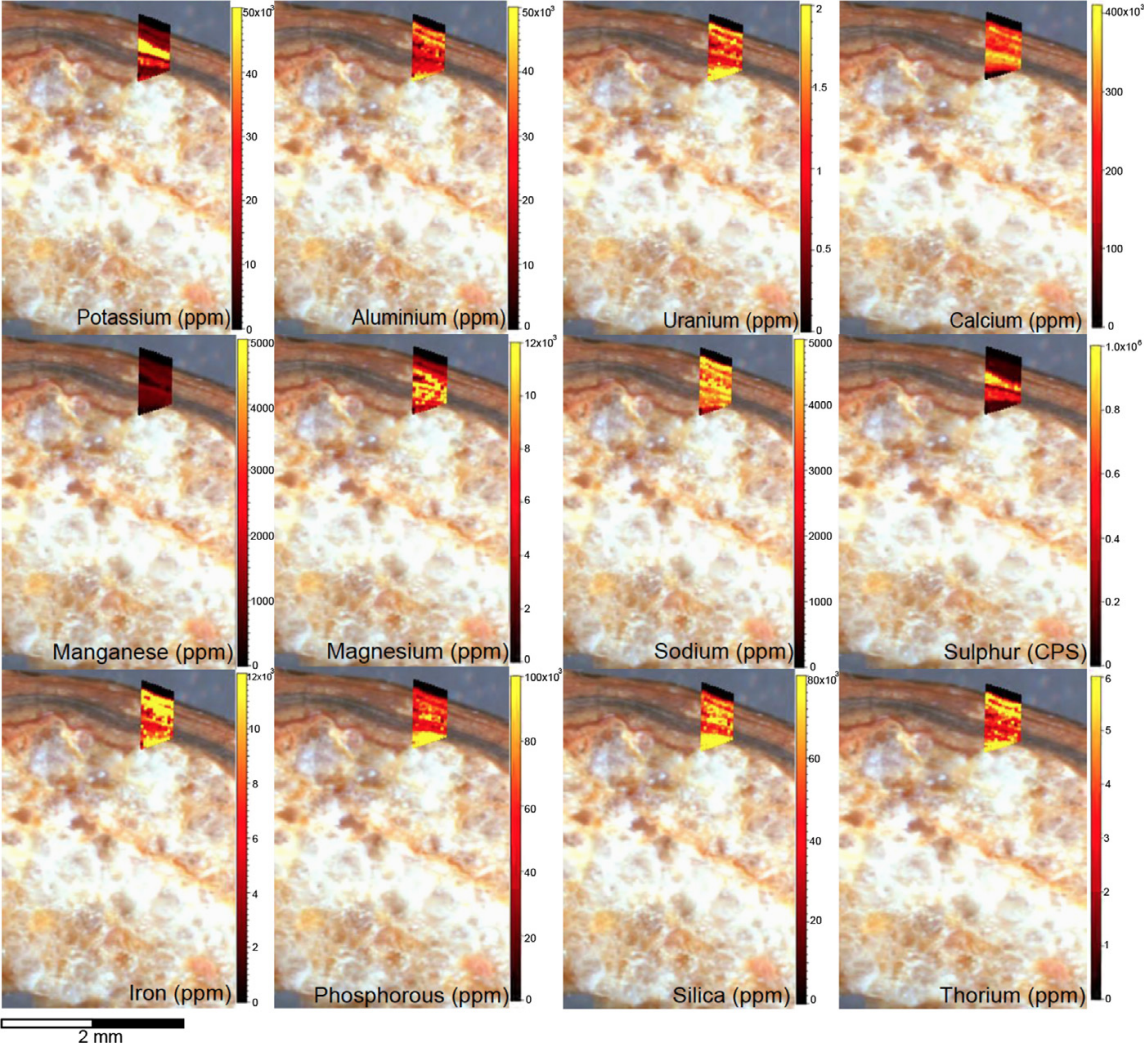
LA-ICP-MS trace element scans of floor glaze sample H309. The scans display the distinct layering present in this accretions characteristic of this mineral system.

**Fig. 13:**
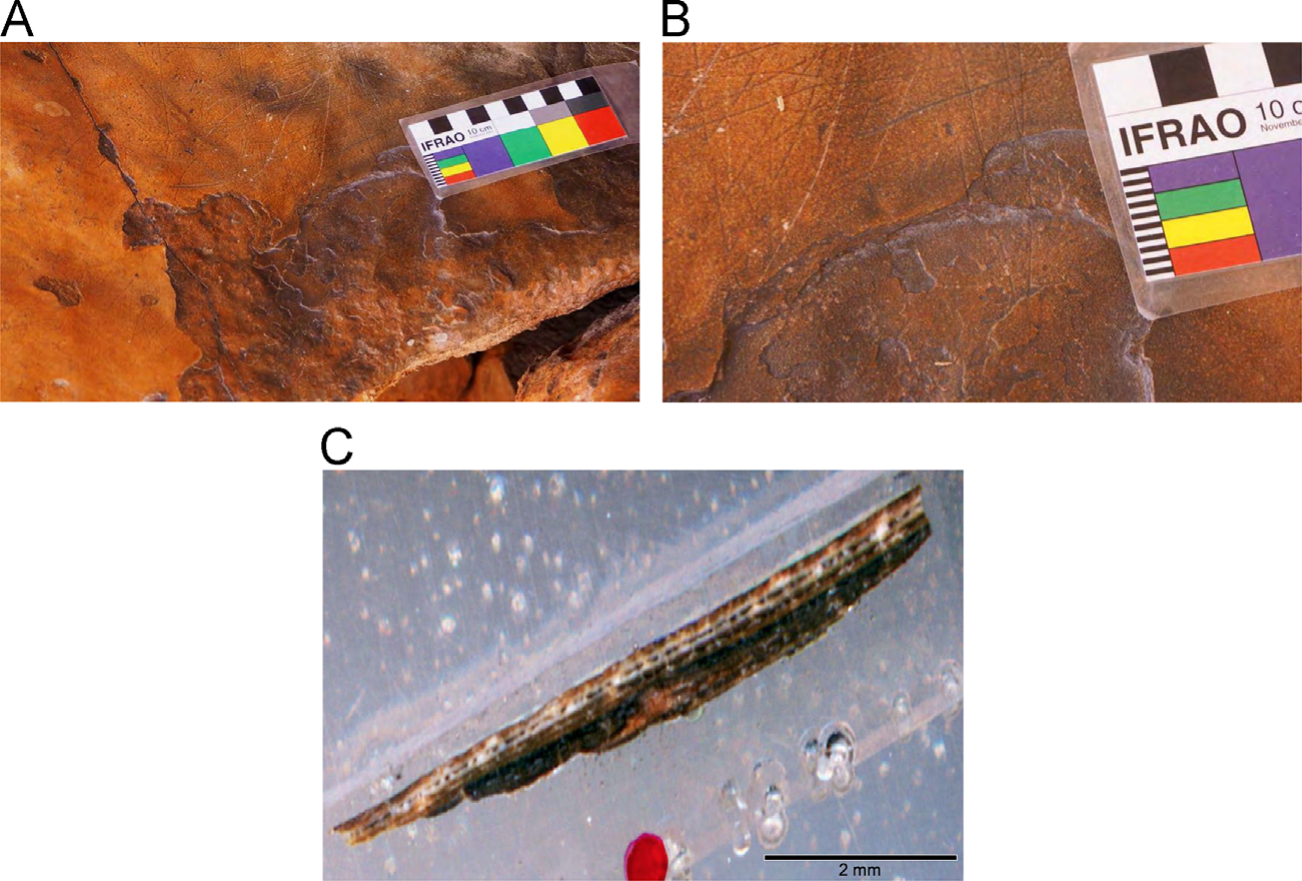
**(A)** Collection of sample H347 from a floor glaze mineral system on a boulder in site KGRD002 in the King George River region, **(B)** Floor glaze displaying distinctive smooth and dark coloured surface of accretion sample H347 prior to removal, **(C)** Cross sectioned mount of floor glaze accretion sample H347 displaying an internal stratigraphy of thin continuous layers.

**Fig. 14:**
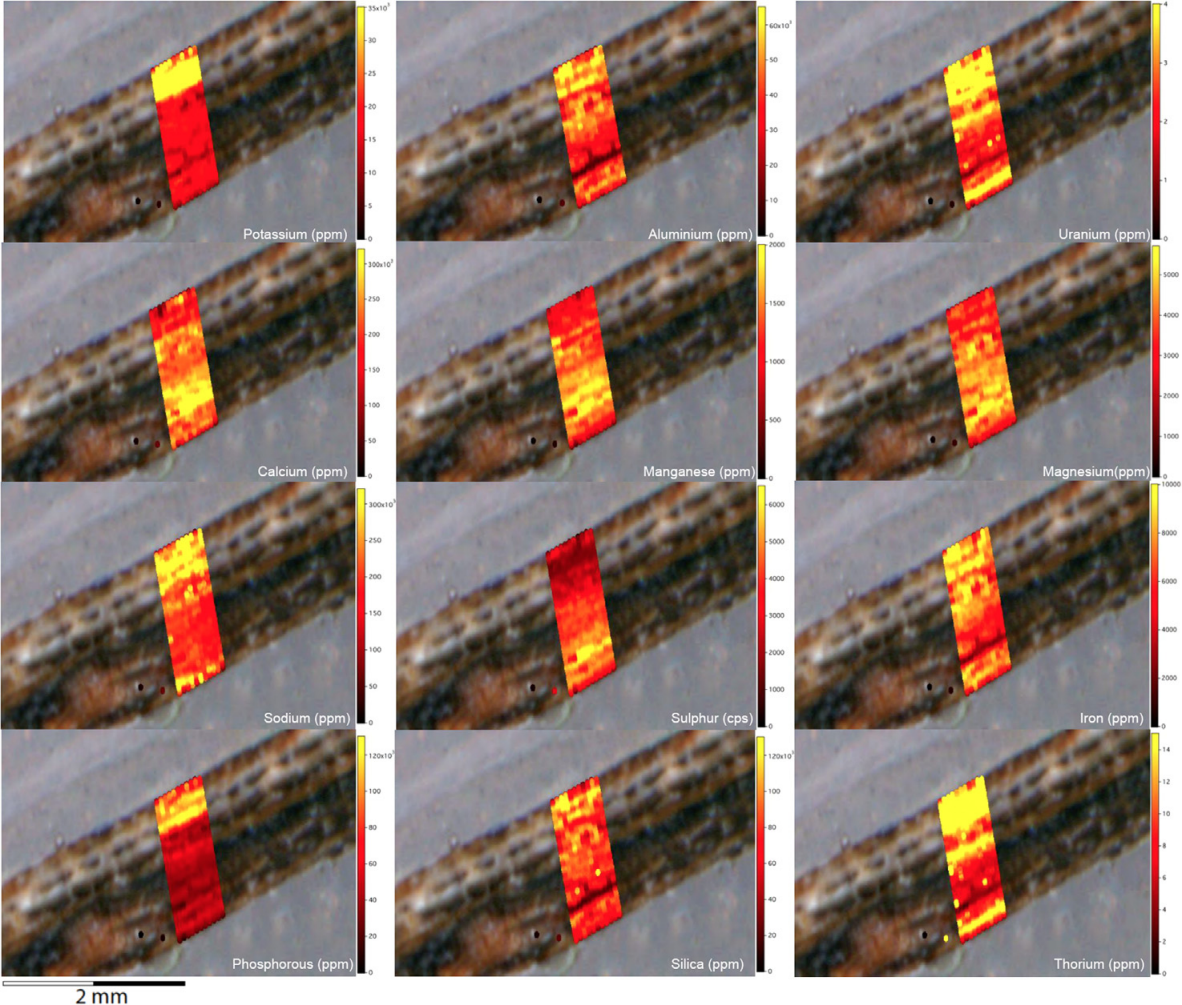
LA-ICP-MS trace element scans of polychrome floor glaze sample H347. The scans display the distinct layering present in this accretion, characteristic of this mineral system.

**Fig. 15:**
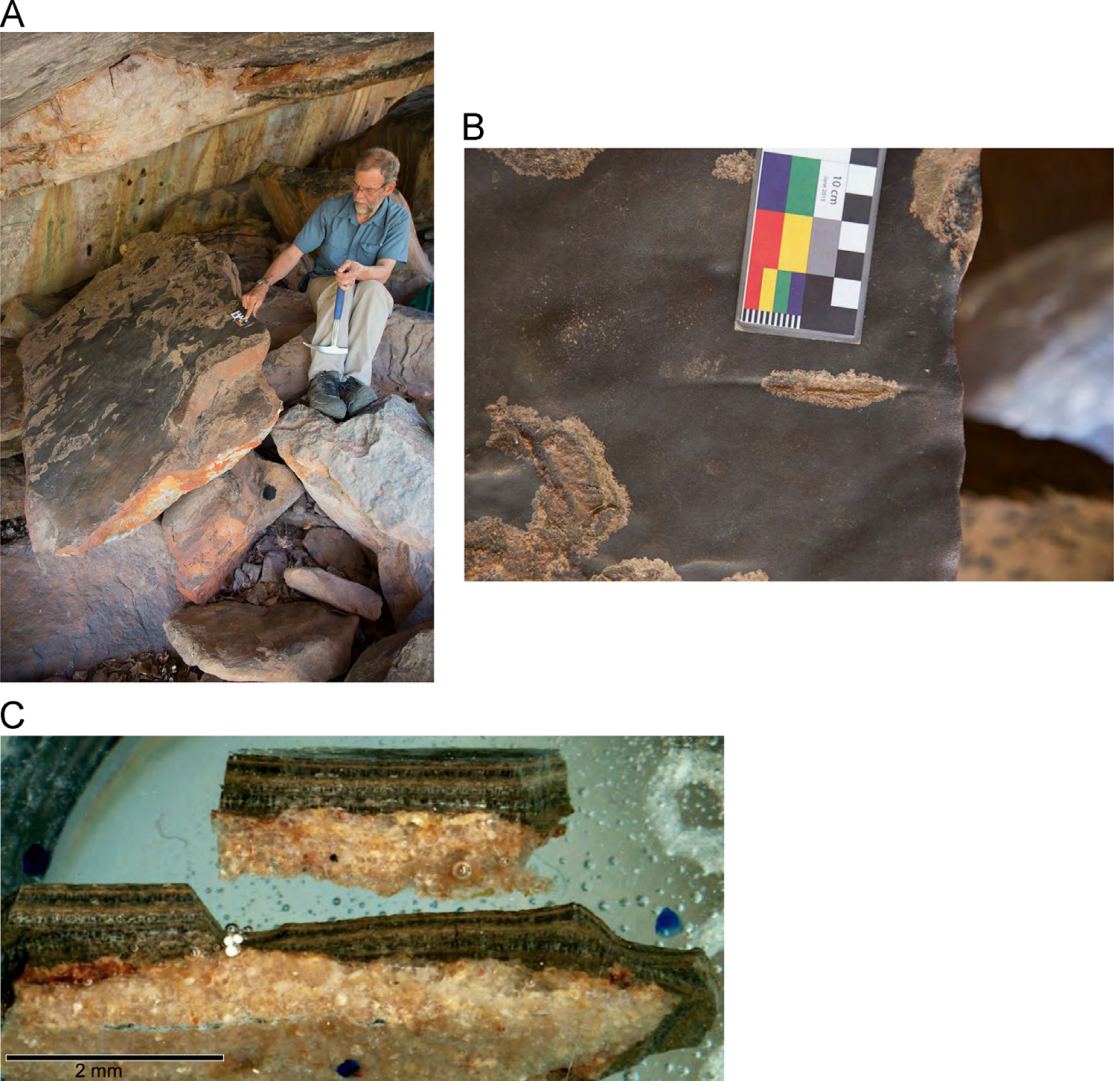
**(A)** Collection of sample H076 from a floor glaze mineral system on a boulder in site DRY017 in the Drysdale River National Park region, **(B)** Floor glaze displaying smooth, dark surface of accretion sample H076 displaying a detailed internal stratigraphy.

**Fig. 16:**
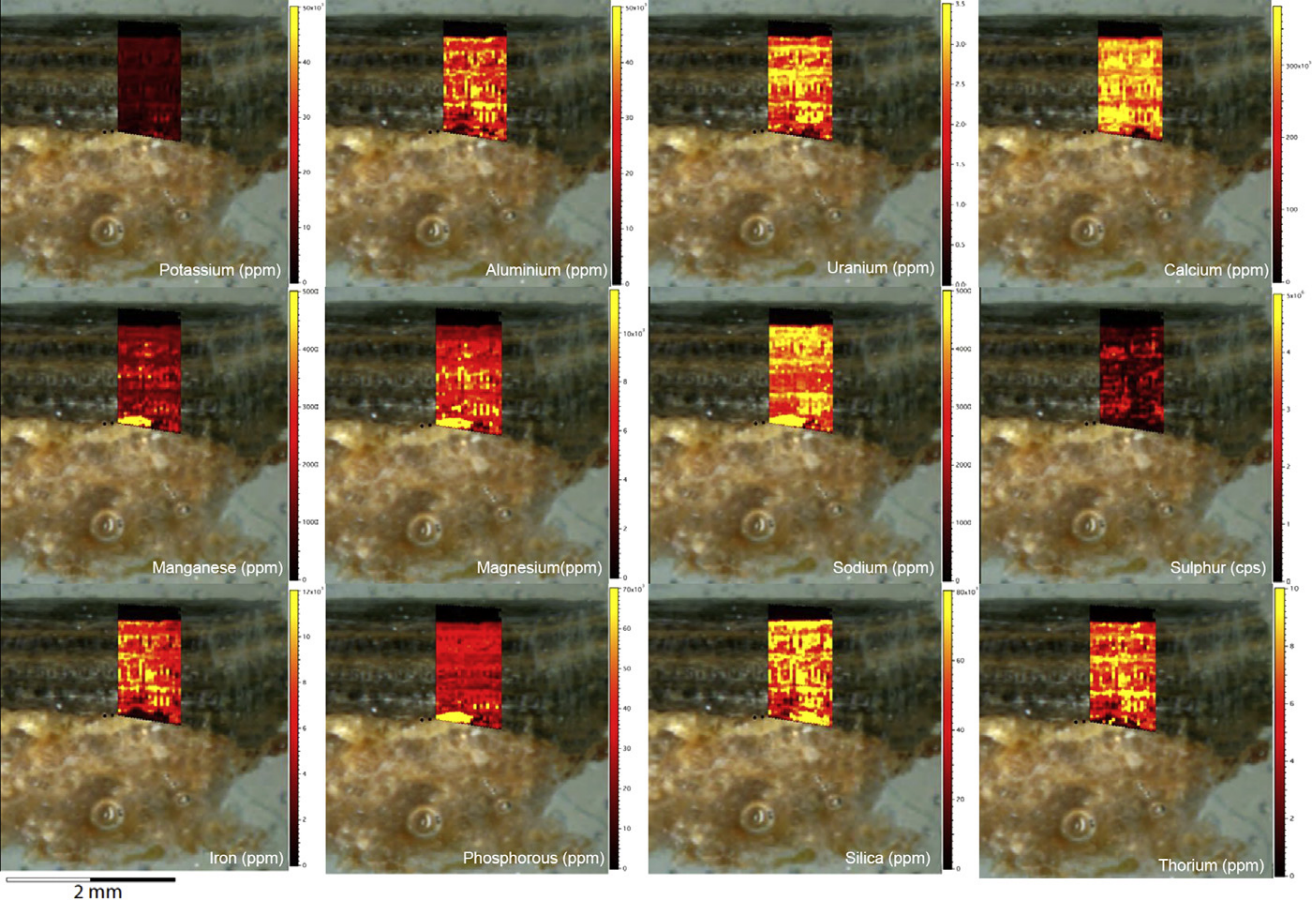
LA-ICP-MS trace element scans of floor glaze sample H076. The scans display the distinct and continuous layering present in the accretions characteristic of this mineral system.

**Fig. 17:**
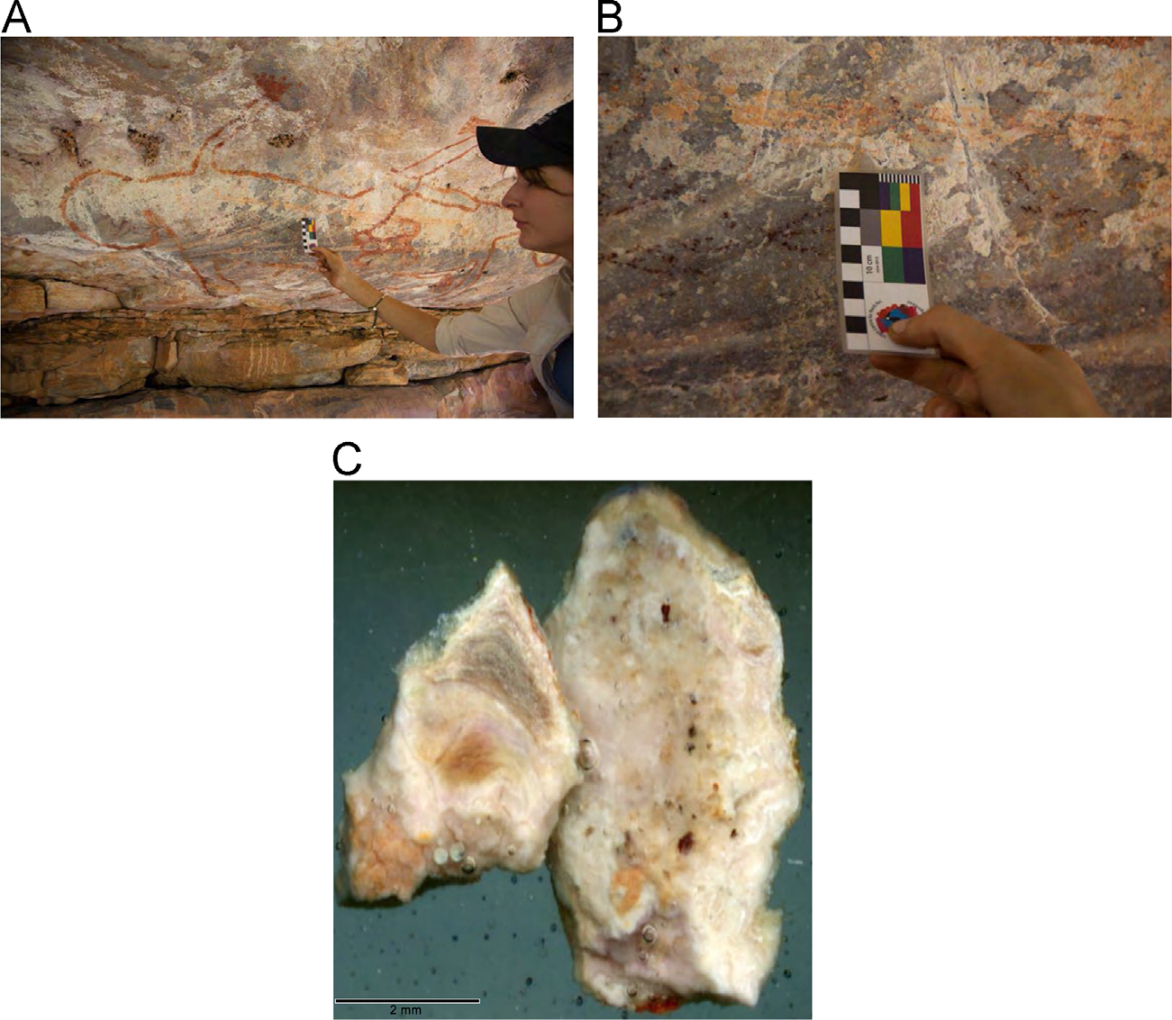
**(A)** Collection of sample H038 from a dispersed wall coating mineral system on the back wall of site DRY013 in the Drysdale River National Park region, **(B)** Dispersed wall coating displaying dispersed and nodular occurrence of sample H038 prior to removal, **(C)** Cross sectioned mount of dispersed wall coating accretion sample H038.

**Fig. 18:**
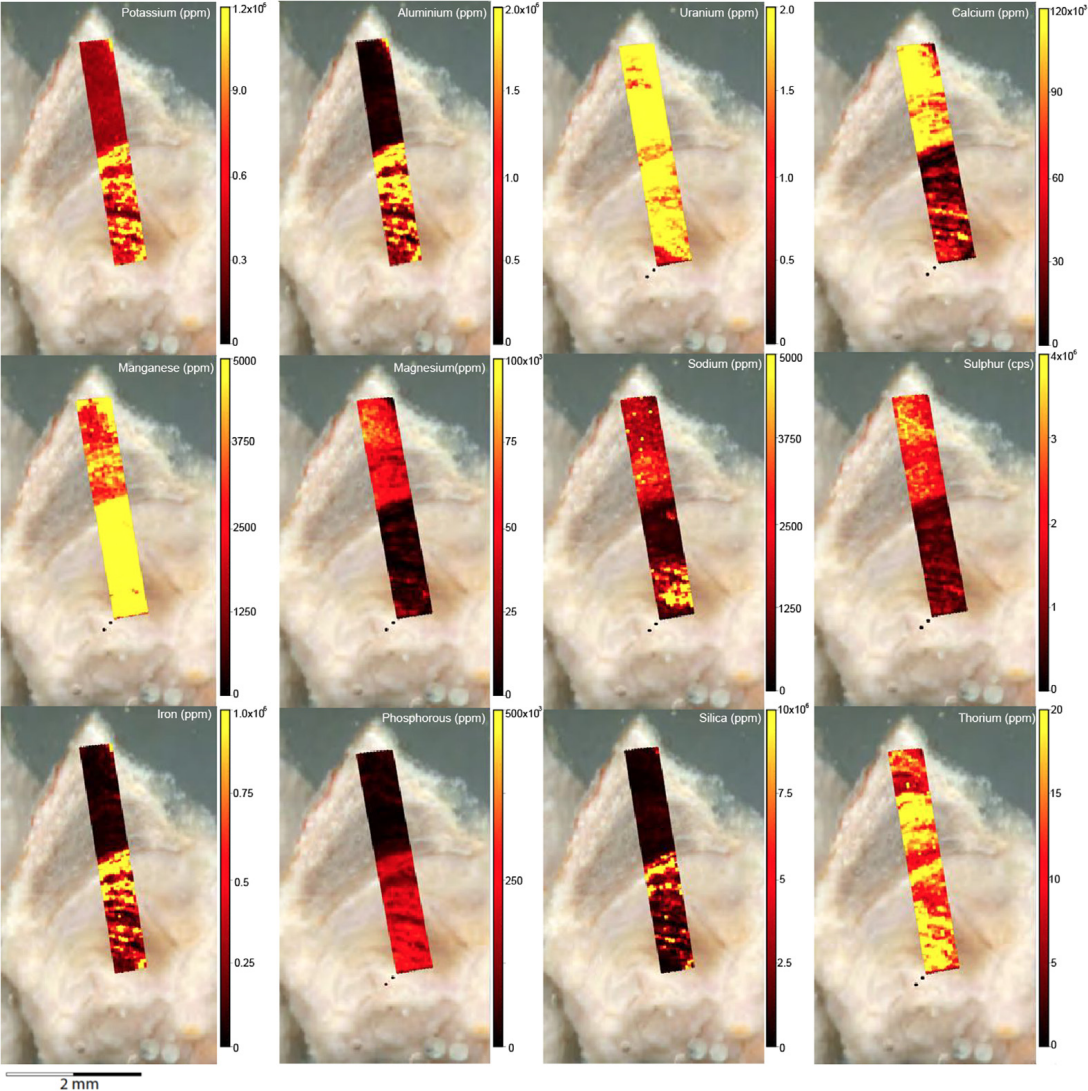
LA-ICP-MS trace element scans of dispersed background coating sample H038.

**Fig. 19:**
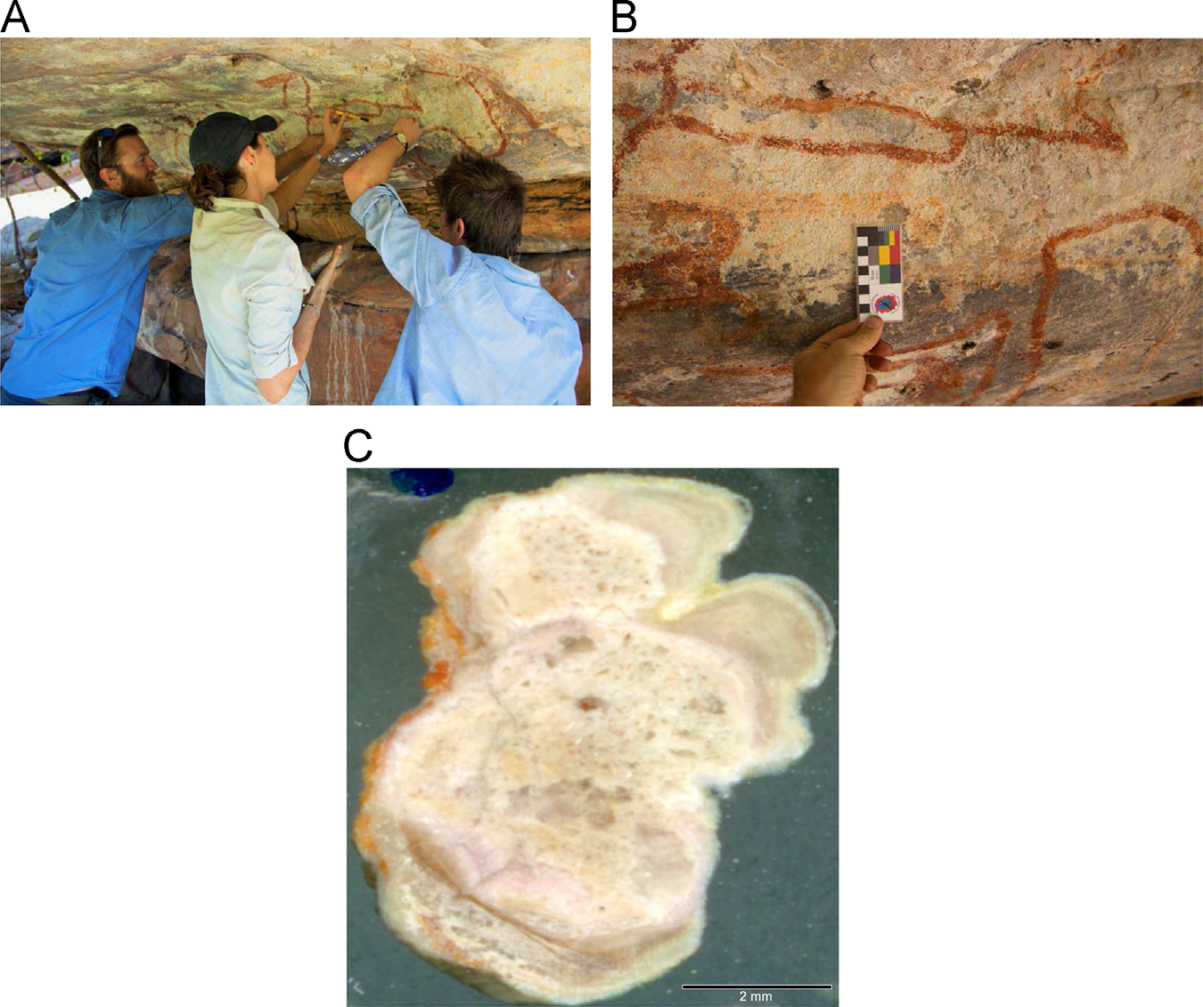
**(A)** Collection of sample H040 from a dispersed wall coating mineral system on the back wall of a shelter in site DRY13 in the Drysdale River National Park region, **(B)** Dispersed wall coating providing a backdrop for rock art in the shelter. The accretion is an accumulation of white, friable nodules **(C)** Cross sectioned mount of dispersed wall coating accretion sample H040 displaying a generally homogenous interior with small areas of internal layering.

**Fig. 20:**
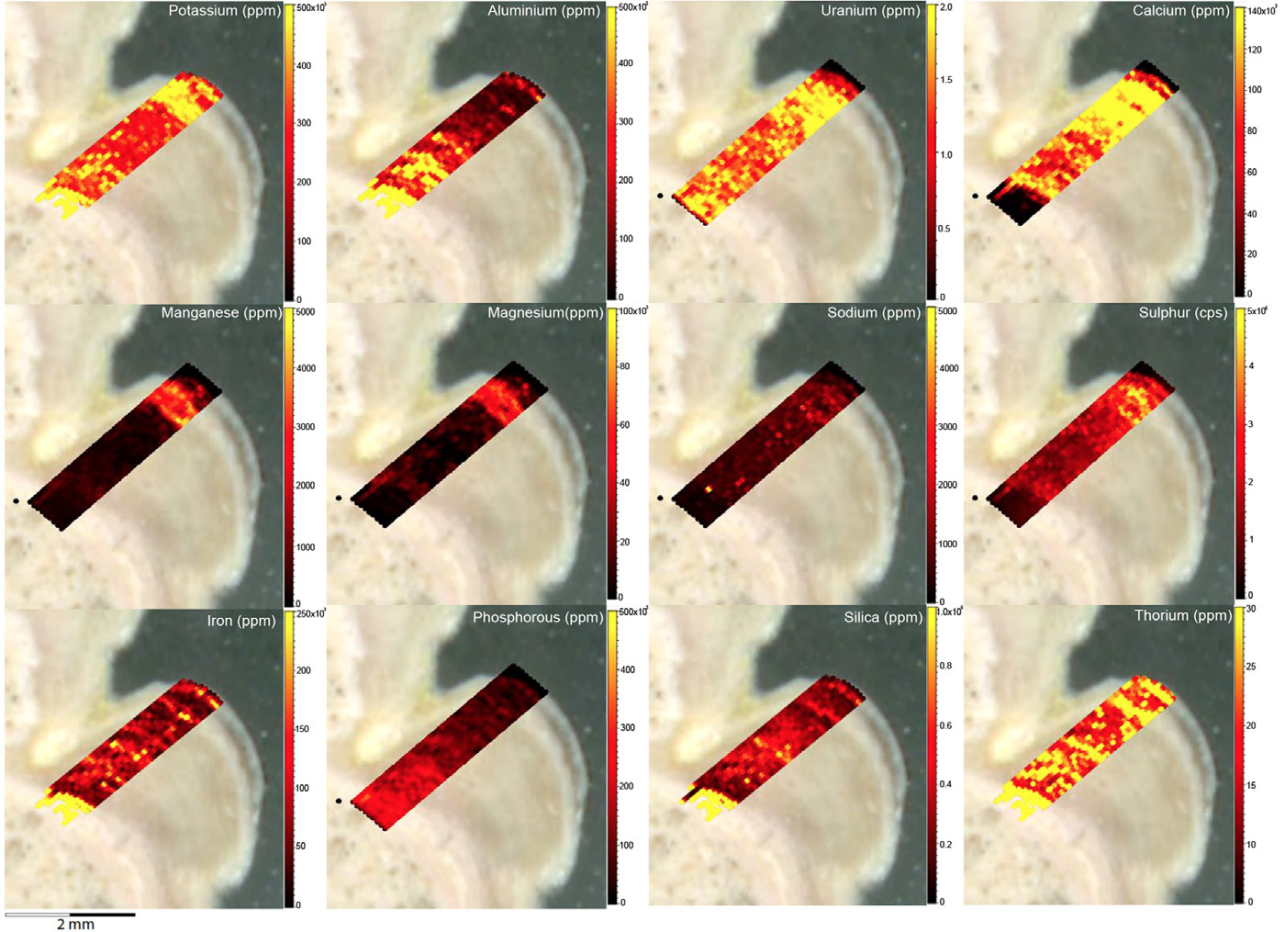
LA-ICP-MS trace element scans of dispersed wall coating sample H040. The scans display a small area of layering present in an otherwise homogenous sample which is generally characteristic of this mineral system.

**Fig. 21:**
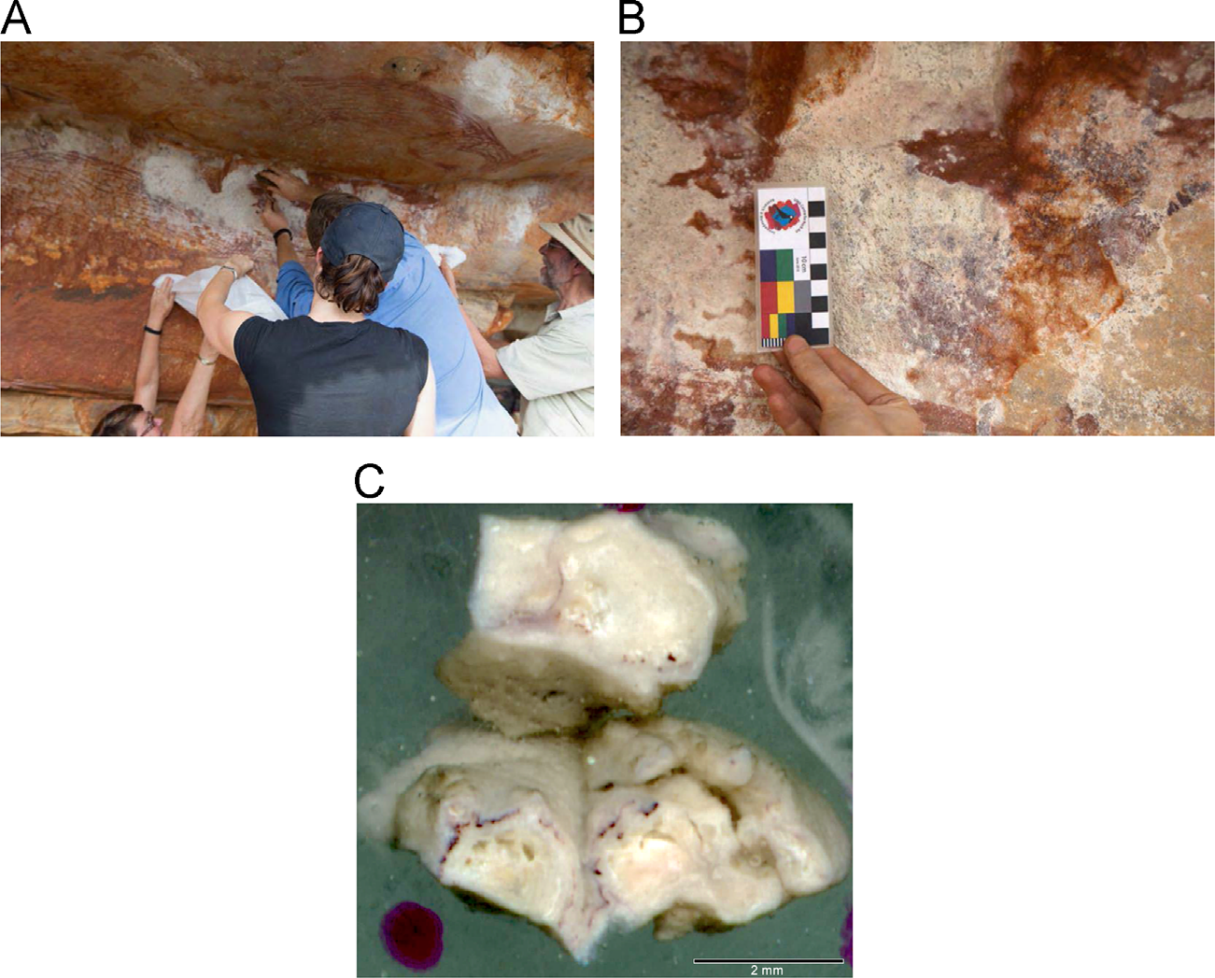
**(A)** Collection of sample H023 from a dispersed wall coating mineral system on the ceiling of site DRY010 in the Drysdale River National Park region, **(B)** Dispersed wall coating displaying a discontinuous, white surface associated with rock art, **(C)** Cross sectioned mount of accretion sample H023.

**Fig. 22:**
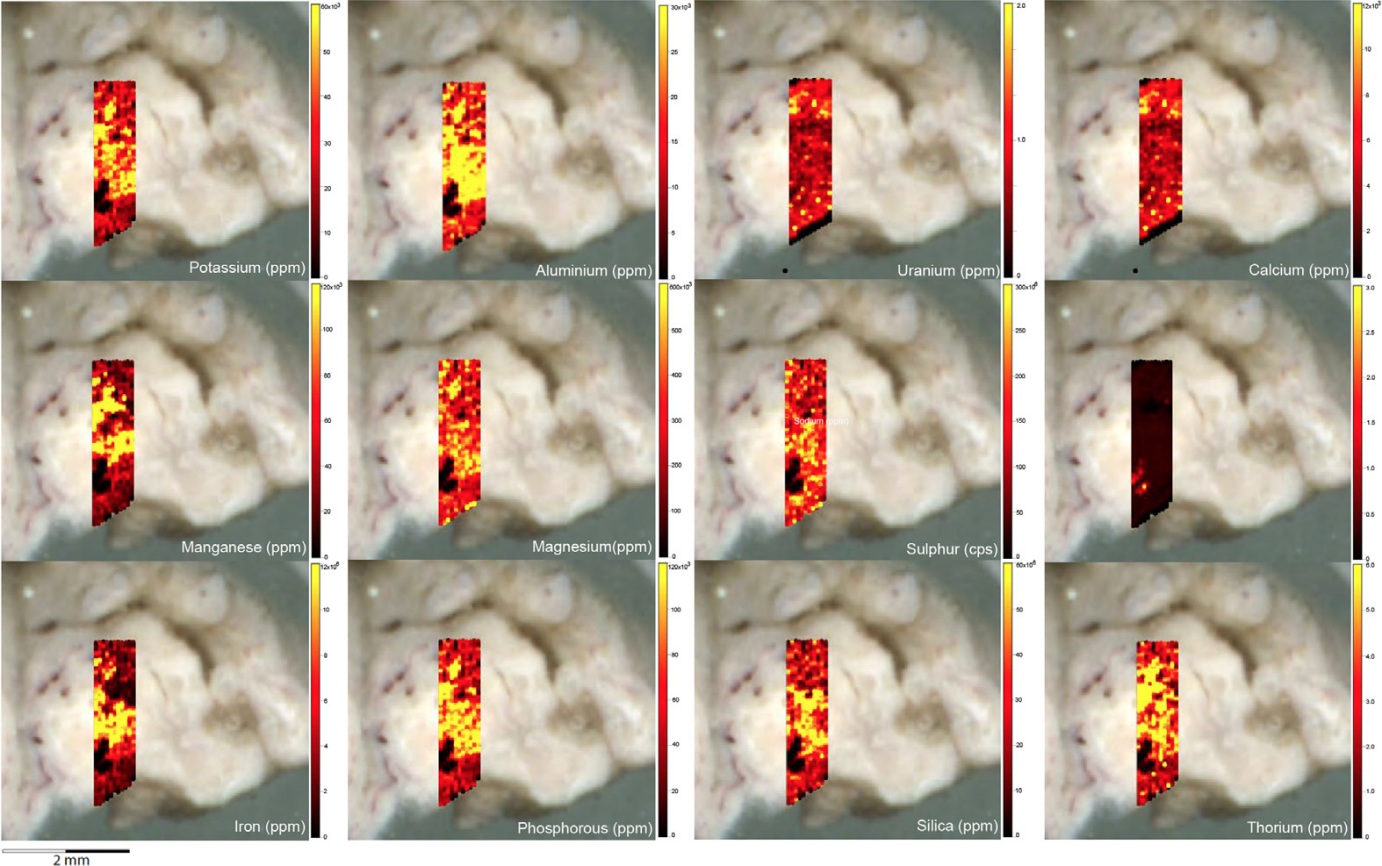
LA-ICP-MS trace element scans of dispersed background coating sample H023.

Laser ablation trace element maps of cross sectioned mineral accretions collected at rock art shelters in the Kimberley display variations in the characteristics of their internal micro-stratigraphies dependent on the mineral system to which they have been assigned (Fig. 1, Fig. 2, Fig. 3, Fig. 4, Fig. 5, Fig. 6, Fig. 7, Fig. 8, Fig. 9, Fig. 10, Fig. 11, Fig. 12, Fig. 13, Fig. 14, Fig. 15, Fig. 16, Fig. 17, Fig. 18, Fig. 19, Fig. 20, Fig. 21, Fig. 22) [1]. The accretions also display differing concentrations of particular elements, which is useful for the assessment of the suitability of each accretion, and in turn, mineral system to particular radiogenic dating techniques. The varying concentration of these different elements also provides information which aids the generation of hypotheses surrounding the formation mechanisms associated with the different mineral systems.

### 1.2 Data from X-ray diffraction analysis

Table 1, Table 2 display the occurrence of a range of sulphate, oxalate and phosphate minerals across the four different mineral systems from a range of sites across the Kimberley. This is a semi-quantitative analysis of the crystalline component of the sample as X-ray Diffraction does not detect any amorphous content that may be present. Table 1 includes data from 44 samples of Polychrome Fringes. Table 2 includes 38 samples from the other three mineral deposition systems; twenty-six samples of Dispersed Wall Coatings, seven Floor Glazes examples and five Silica Stalagmites and Skin samples. Within the mineral systems, samples are grouped geographically into three broad areas. Central Sites are those in the general area of the Drysdale River National Park. Sites in the west are in the Doubtful Bay region and sites in the north are in the King George River area.

**Table 1:** Semi-quantitative X-ray diffraction mineralogical data for 44 Polychrome Fringe accretion samples collected in the Kimberley region of north west Australia.

**Table 2:** Semi-quantitative X-ray diffraction mineralogical data for 26 Dispersed Wall Coating accretion samples, 7 Floor Glazes and 5 Silica Skins/Stalagmites collected in the Kimberley region of north west Australia.

### 1.3 Data from scanning electron microscope analysis

**Fig. 23:**
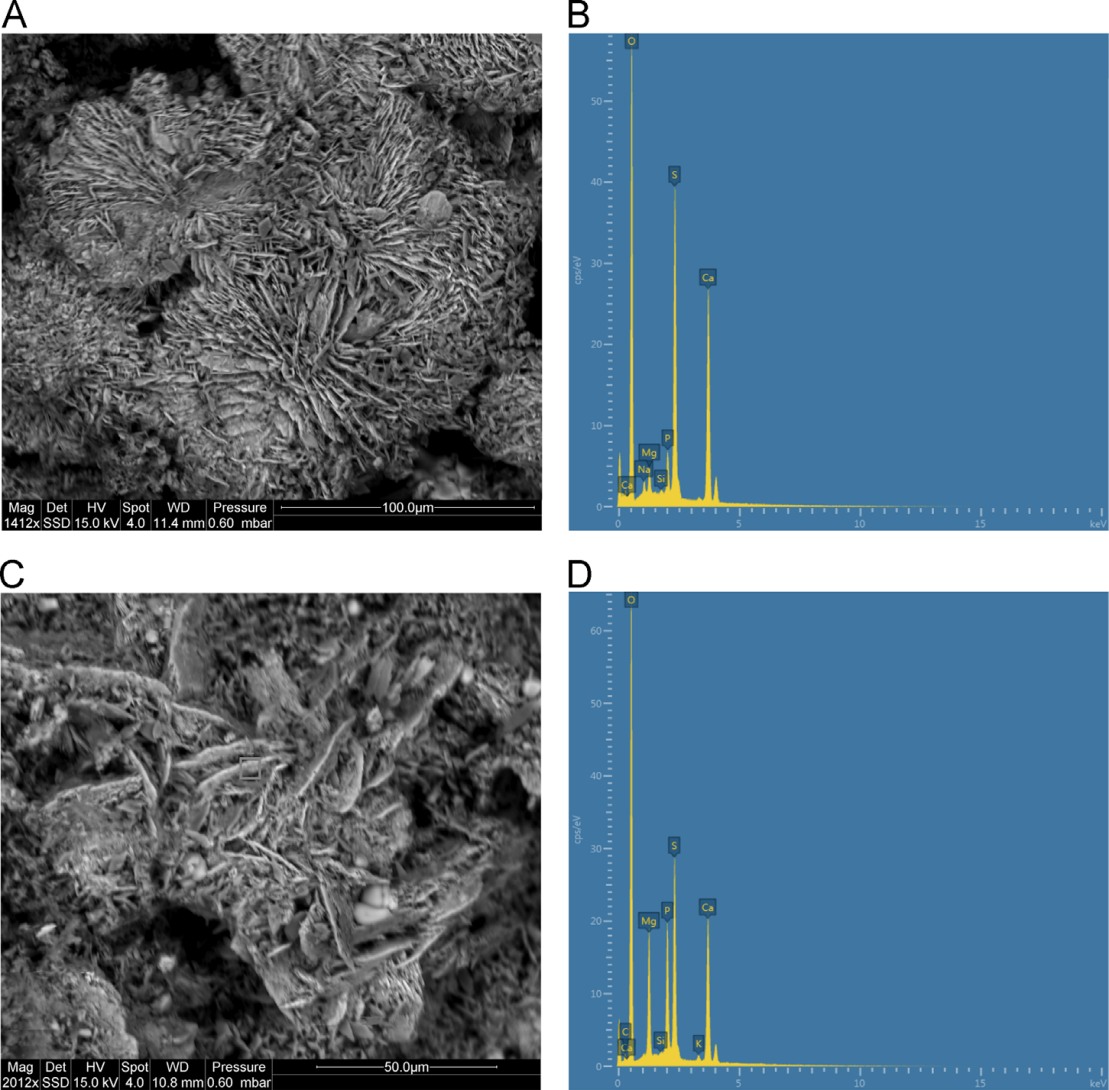
**(A)** SEM image of polychrome accretion sample K12-25 collected in the Drysdale River region, box indicates site of EDS analysis, **(B)** EDS spectra displaying major peaks at O, S and Ca. **(C)** SEM image of polychrome accretion sample K12-25, box indicates site of EDS analysis. **(D)** EDS spectra displaying major peaks at O, Mg, P, S and Ca.

**Fig. 24:**
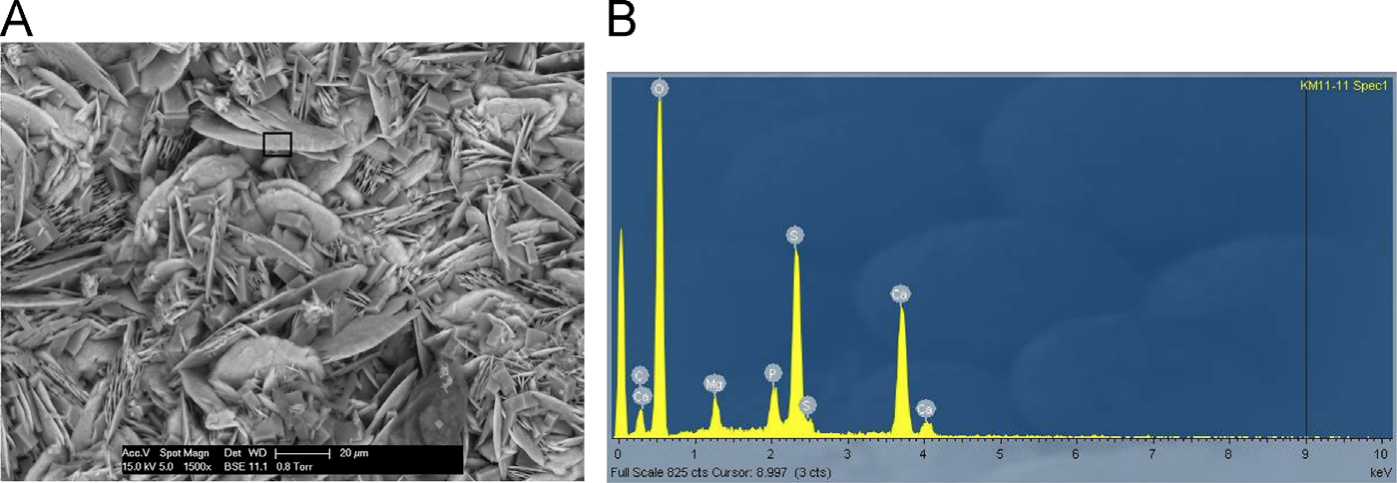
**(A)** SEM image of polychrome accretion sample K11-11 collected in the Drysdale River region, box indicates site of EDS analysis. The image displays the intimate, mixed range of well crystallised minerals. EDS spectra identifies sulphate crystals, however XRD analysis also confirms the presence of oxalate and phosphate minerals including whewellite and newberyite. **(B)** EDS spectra displaying major peaks at O, S and Ca.

**Fig. 25:**
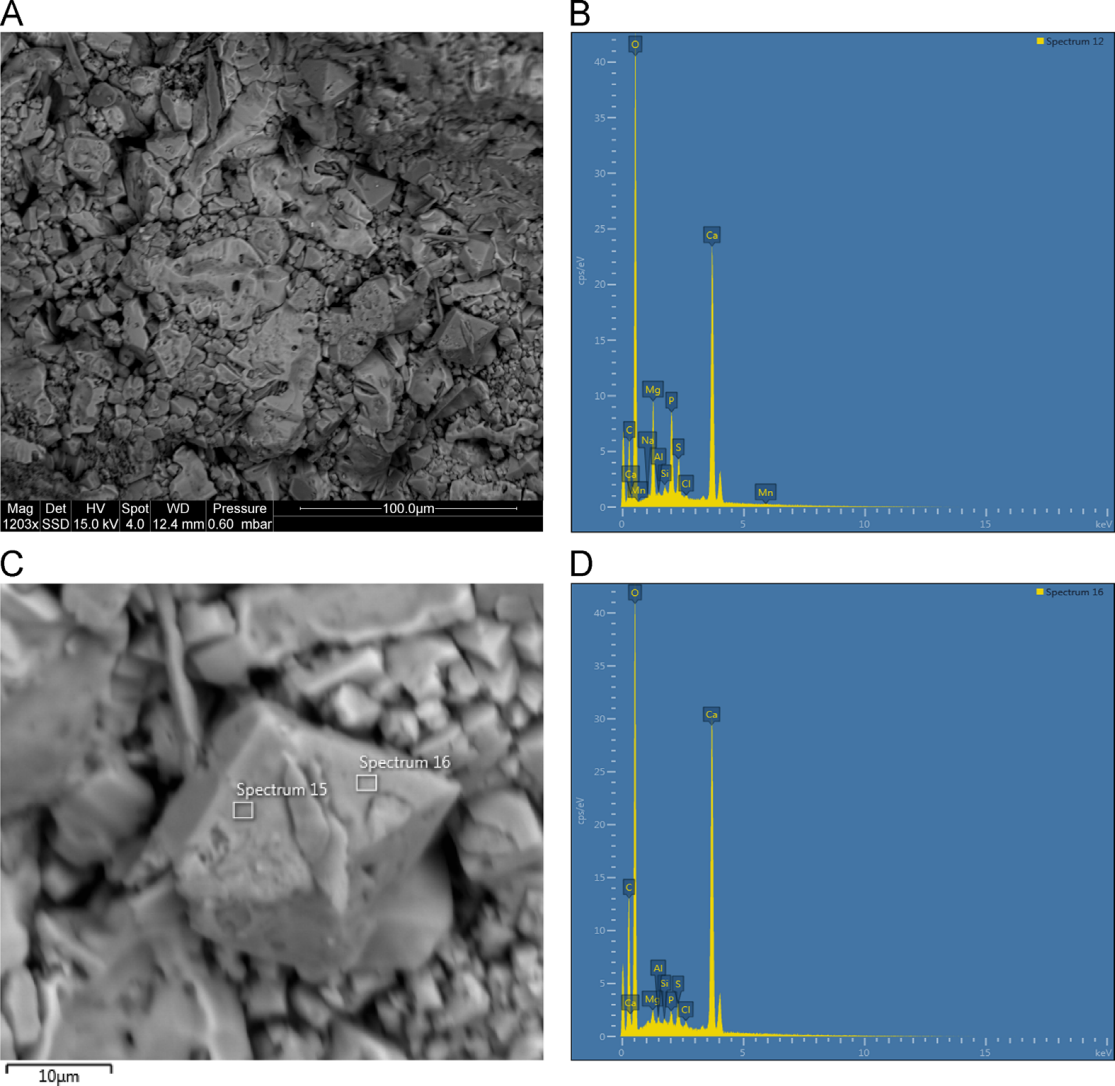
**(A)** SEM image of polychrome accretion sample K12-02 collected in the Drysdale River region, **(B)** EDS spectra displaying major peaks at C, O, Mg, P and Ca, box indicates oxalate crystal in figure C. **(C)** SEM image of characteristically shaped oxalate crystal in polychrome accretion sample K12-02. White boxes indicate site of EDS analysis (spectrum 16). **(D)** EDS spectra displaying major peaks at C, O and Ca.

**Fig. 26:**
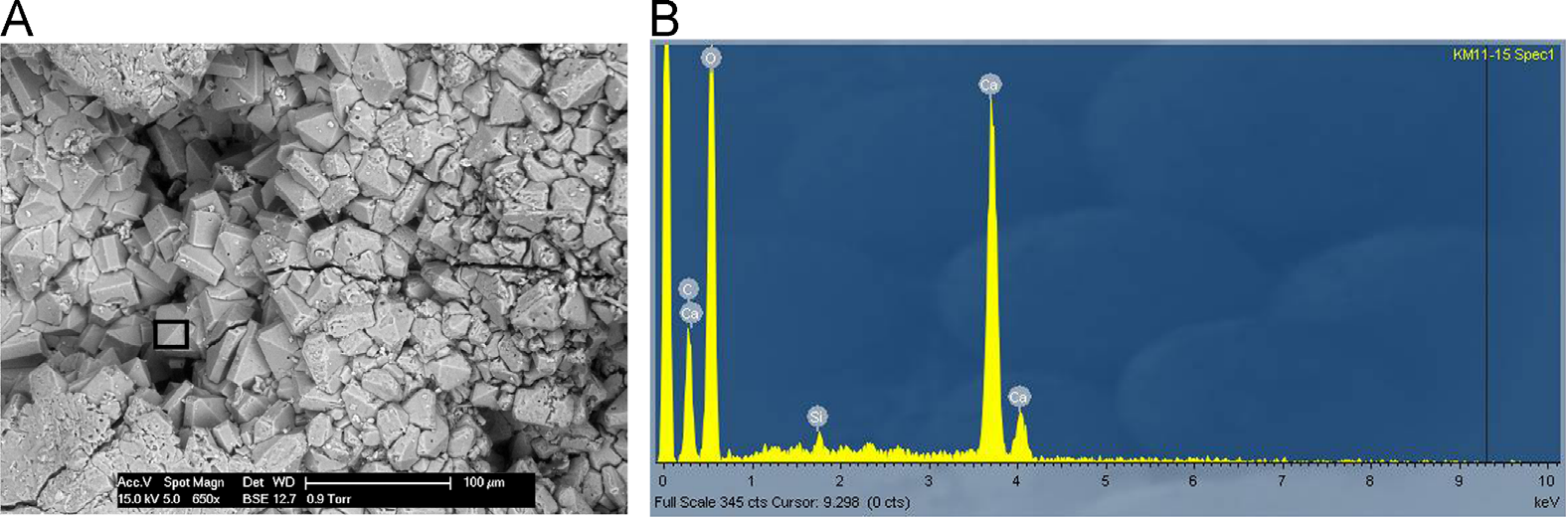
**(A)** SEM image of polychrome accretion sample K11-15 collected in the Drysdale river region, box indicates site of EDS analysis. The image displays well crystallised minerals displaying the characteristic shape of calcium oxalate minerals. **(B)** EDS spectra displaying major peaks at C, Ca and O.

**Fig. 27:**
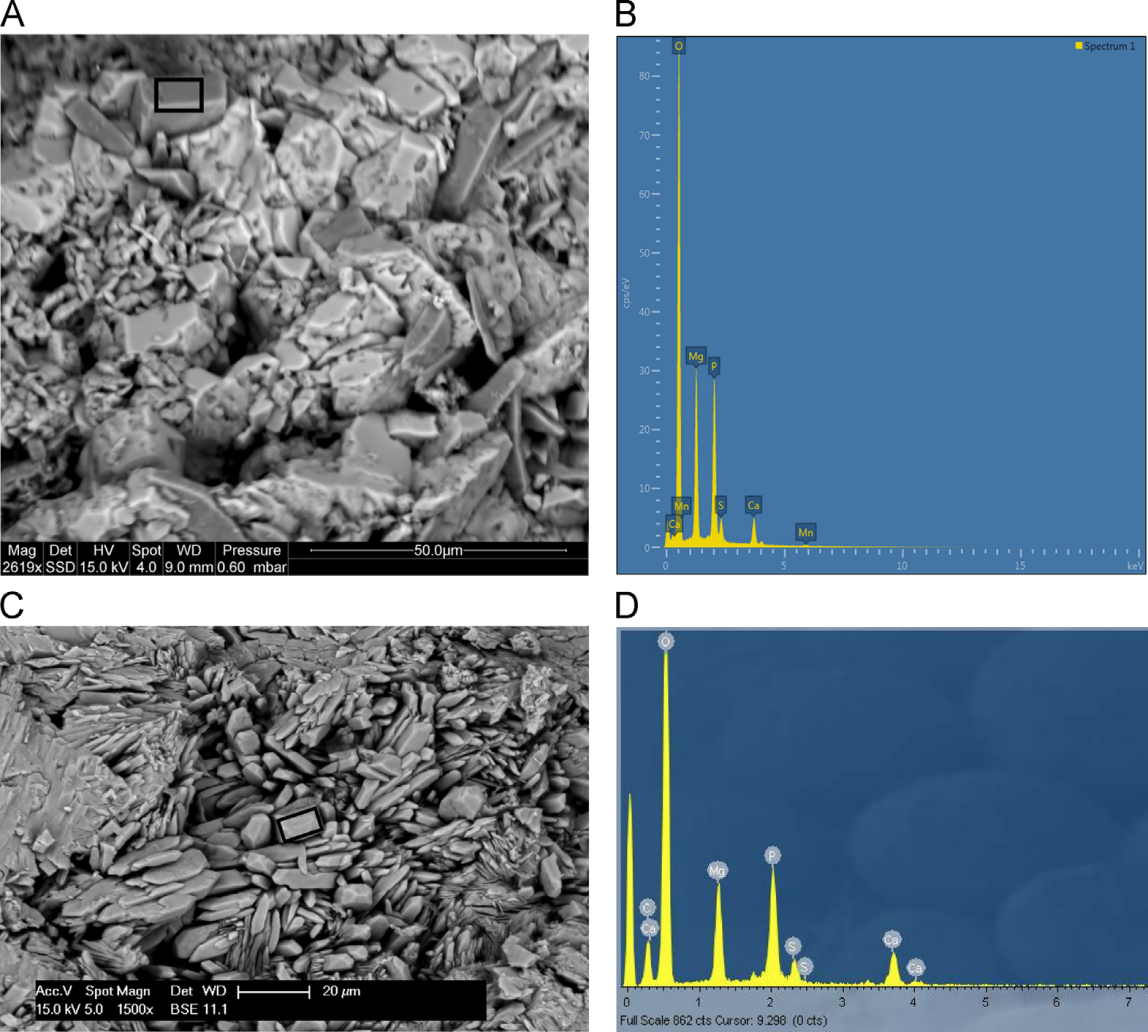
**(A)** SEM image of polychrome accretion sample K12-02 collected in the Drysdale river region. Black box indicates site of EDS analysis. **(B)** EDS spectra displaying major peaks at O, Mg and P, suggesting the presence of magnesium phosphate minerals. **(C)** SEM image of polychrome accretion sample OM14-14 collected in the King George River region, black box indicates site of EDS analysis. **(D)** EDS spectra displaying major peaks at C, Ca, O, Mg and P.

**Fig. 28:**
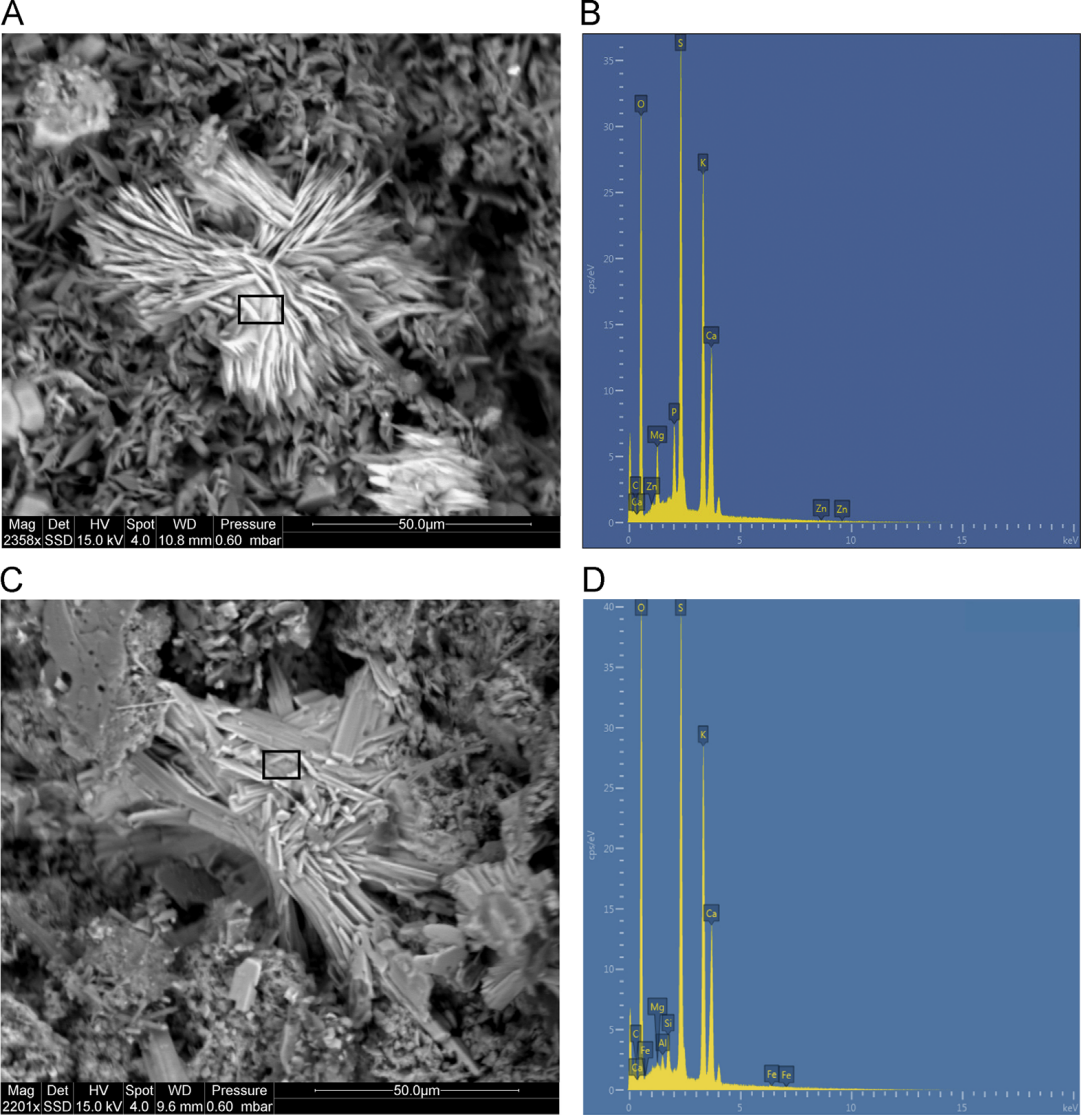
**(A)** SEM image of polychrome accretion sample K12-25 collected in the Drysdale river region. Black box indicates site of EDS analysis. **(B)** EDS spectra displaying major peaks at O, S, K and Ca, suggesting the presence of potassium-calcium sulphate minerals. **(C)** SEM image of polychrome accretion sample K12-40 collected in the Drysdale River region, black box indicates site of EDS analysis. **(D)** EDS spectra displaying major peaks at O, S, K and Ca.

Backscattered electron (BSE) imaging data (Fig. 23, Fig. 24, Fig. 25, Fig. 26, Fig. 27, Fig. 28) demonstrates the presence of well crystallised and intimately mixed phosphate, oxalate and sulphate minerals in several mineral accretion examples from Kimberley rock shelters. SEM-EDS data from the mineral accretions indicate the presence of the oxalate mineral whewellite, sulphate mineral gypsum and phosphate mineral newberyite as well as suggesting the presence of rarer potassium calcium sulphate minerals [1].

## 2 Experimental design, materials and methods

### 2.1 Study area description

Mineral accretions were collected from rock shelter walls hosting rock art in several locations across the Kimberley region of northern Western Australia (See Fig. 1 of [1]). The site codes are given in figure captions and relate to samples documented in an access-controlled archaeological site catalogue held at The University of Western Australia's Centre for Rock Art Research + Management.

### 2.2 X-ray diffraction

Characterisation of accretion mineralogy was predominantly established using a Bruker D8 Advance x-ray powder diffractometer (XRD) with Ni-filtered Cu kα radiation (1.54 Å) at the University of Melbourne Materials Characterisation and Fabrication Platform. Data were collected between 5–85° 2θ, with a step size of 0.02° and a scan rate of 1.0 s per step. An incident beam divergence of 0.26° was used with a 2.5° soller slit in the diffracted beam. The sample was spun at 15 revolutions per minute. The background was fixed manually. Following measurement, phase identification was completed using Materials Data, Inc., Jade 9.3 and Bruker EVA software with the ICDD PDF-2 and PDF-4 databases with key mineral phases established for each sample using standard search-matching procedures. Several samples were also analysed at The Melbourne Museum using a Phillips X’Pert PRO XRD system traversing a scattering angle of 75°, coupled to an X’Pert Data Collector with X’Pert HighScore search-match software. Data is presented in Table 1, Table 2.

### 2.3 Laser-ablation trace element mapping

Elemental distribution maps were used to analyse the internal structures of the accretion samples. Maps were produced using LA-ICP-MS analyses, performed on an Agilent 7700x quadrupole mass spectrometer, coupled to a Lambda Physik Compex UV 193 nm excimer laser system at the University of Melbourne employing a S-155 ablation cell. Samples were ablated under helium with an argon carrier gas. Prior to laser ablation analysis, samples were mounted in a 'freeform' sample holder and imaged at high resolution on a flatbed scanner. The resulting images, referenced to the coordinate system of the ablation cell, could then be used as a base layer upon which to overlay the laser ablation concentration ‘maps’. The latter were produced by analysing the material liberated from a series of parallel ablation tracks across the sample surface, oriented perpendicular to the growth banding. The analysis protocol utilised a scan speed of 40 µm s^-^^1^, with a spot size of 40 µm, pulse rate of 10 Hz, and laser fluence of ~2.5 J/cm^−2^. NIST SRM 612 was used as the primary calibration material. In all laser analyses reported herein, the elements for which data were acquired are Al, Ca, Ce, Fe, Mg, Mn, P, S, Sr, Si, Th and U with an estimated precision of elemental concentrations of ca <5%. All data were reduced using Iolite software [2] with data deconvolution as described in [3]. Data are presented in Fig. 1, Fig. 2, Fig. 3, Fig. 4, Fig. 5, Fig. 6, Fig. 7, Fig. 8, Fig. 9, Fig. 10, Fig. 11, Fig. 12, Fig. 13, Fig. 14, Fig. 15, Fig. 16, Fig. 17, Fig. 18, Fig. 19, Fig. 20, Fig. 21, Fig. 22.

### 2.4 Scanning electron microscopy

The distribution and morphology of minerals within accretions were established using both a Quanta FEG 200 ESEM and a Phillips FEI XL30 environmental scanning electron microscope (ESEM) equipped with an OXFORD INCA energy-dispersive x-ray spectrometer (EDS) at the University of Melbourne. The instrument has a tungsten filament electron source with a beam of 15 kV and spot size 6 and was operated in high vacuum mode with a gold coating used to render the samples conductive. The EDS system uses a liquid-nitrogen cooled Si-Li detector with an area of 10 mm^2^ and an ATW2 thin detector window allowing collection of x-rays between B and U. Valuable information was obtained from the morphology of crystalline forms and the elemental composition of each constituent was determined using EDS spot analysis. Data are presented in Fig. 23, Fig. 24, Fig. 25, Fig. 26, Fig. 27, Fig. 28.
